# The intracellular C-terminus confers compartment-specific targeting of voltage-gated calcium channels

**DOI:** 10.1016/j.celrep.2024.114428

**Published:** 2024-07-11

**Authors:** Morven Chin, Pascal S. Kaeser

**Affiliations:** 1 Department of Neurobiology, Harvard Medical School, Boston, MA 02115, USA; 2 Lead contact

## Abstract

To achieve the functional polarization that underlies brain computation, neurons sort protein material into distinct compartments. Ion channel composition, for example, differs between axons and dendrites, but the molecular determinants for their polarized trafficking remain obscure. Here, we identify mechanisms that target voltage-gated Ca^2+^ channels (Ca_V_s) to distinct subcellular compartments. In hippocampal neurons, Ca_V_2s trigger neurotransmitter release at the presynaptic active zone, and Ca_V_1s localize somatoden-dritically. After knockout of all three Ca_V_2s, expression of Ca_V_2.1, but not Ca_V_1.3, restores neurotransmitter release. We find that chimeric Ca_V_1.3s with Ca_V_2.1 intracellular C-termini localize to the active zone, mediate synaptic vesicle exocytosis, and render release sensitive to Ca_V_1 blockers. This dominant targeting function of the Ca_V_2.1 C-terminus requires the first EF hand in its proximal segment, and replacement of the Ca_V_2.1 C-terminus with that of Ca_V_1.3 abolishes Ca_V_2.1 active zone localization and function. We conclude that Ca_V_ intracellular C-termini mediate compartment-specific targeting.

## In brief

Voltage-gated Ca^2+^ channel subtypes are differentially sorted into the soma, dendrites, and axon of a neuron. Chin et al. find that the intracellular C-termini of Ca_V_1 and Ca_V_2 channels are key determinants for compartment targeting and report an essential trafficking role for the first EF hand of Ca_V_2.1.

## Graphical Abstract

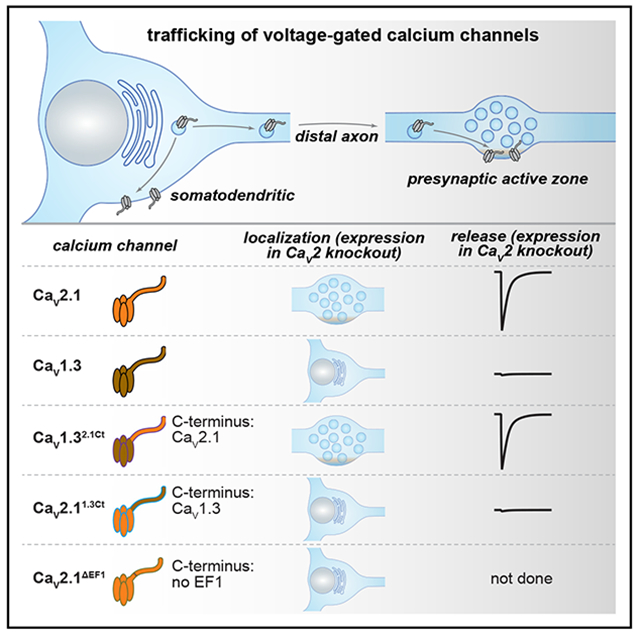

## INTRODUCTION

Neurons are polarized cells with a defined signaling directionality from dendrites to soma to axon.^1^ To achieve this morphological and functional polarization, neurons sort protein material into specific subcellular compartments.^2,3^ Voltage-gated Ca^2+^ channels (Ca_V_s), which couple electrical activity to intracellular Ca^2+^ signaling, are a prototypical example of sorting specificity. They are a large protein family, and individual members localize to distinct subcellular domains in the dendrites, soma, and axon.^4,5^ However, Ca_V_ subtypes exhibit limited sequence differences, and the molecular determinants that target Ca_V_s to specific compartments remain elusive.

Ca_V_s are defined by their pore-forming Ca_V_α1 subunit, and their expression, trafficking, and function are modulated by Ca_V_β subunits and Ca_V_α2δ proteins.^4-7^ Vertebrate Ca_V_α1 subunits are encoded by ten genes classified into Ca_V_1 (Ca_V_1.1–1.4, L-type), Ca_V_2 (Ca_V_2.1–2.3, P/Q-, N-, and R-type), and Ca_V_3 (Ca_V_3.1–3.3, T-type) channels. Most Ca_V_s are co-expressed in central neurons. Ca_V_1.2 and Ca_V_1.3 have important roles in the somatodendritic compartment, and their activity there regulates gene transcription^8-11^ and modulates neuronal firing directly and through Ca^2+^-activated K^+^ channels.^12-16^ In presynaptic nerve terminals, Ca_V_2.1 (P/Q-type) and Ca_V_2.2 (N-type) channels are the primary Ca^2+^ sources for synaptic vesicle release.^17-20^ They are recruited to a specialized release apparatus, the active zone, where they are tethered near fusion-competent vesicles.^21-25^ This organization couples action potential-induced Ca^2+^ entry to vesicular release sites for triggering of neurotransmitter exocytosis. Overall, Ca_V_s contribute to diverse cellular processes, and their functions are directly tied to their subcellular localization.

The mechanisms that distinguish Ca_V_1s from Ca_V_2s and sort them into the somatodendritic and axonal compartments, respectively, remain unclear. Starting from their primary site of synthesis in the soma, Ca_V_s likely undergo a series of interactions that target each subtype to its respective subcellular domain.^2,26^ However, Ca_V_s are highly similar in structure,^5,27,28^ and notable overlap exists within the Ca_V_1 and Ca_V_2 interactome. For example, interactions with Ca_V_β, Ca_V_α2δ, and calmodulin have been implicated in Ca_V_ trafficking,^29-36^ but these proteins interact indiscriminately with Ca_V_1s and Ca_V_2s and are thus unlikely to encode specific sorting information.

The intracellular Ca_V_ C-termini might mediate targeting specificity. Ca_V_ C-termini include a proximal segment with two EF hands and an IQ motif, and a distal segment containing binding sites for scaffolding proteins (Figures S1A and S1B). The Ca_V_2 C-terminus binds to the PDZ domain of the active zone protein RIM, and it contains a proline-rich sequence (which is also present in Ca_V_1s) that binds to RIM-binding protein (RIM-BP).^24,37,38^ These distal C-terminal sequences help tether Ca_V_2s to the presynaptic active zone.^20,24,39-44^ Analogous sequences in Ca_V_1.3 bind to the postsynaptic scaffold Shank, and overall, Ca_V_1 C-termini support cell surface expression and the assembly of dendritic Ca_V_1 clusters.^45,46^ A Ca_V_2.1-specific sequence stretch between the proximal C-terminus and the RIM-BP binding site contributes redundantly to Ca_V_2.1 localization alongside the C-terminal interactions with RIM and RIM-BP.^20,47^ Important roles for Ca_V_2 C-termini in channel trafficking are further supported by the finding that Ca_V_2.1 is absent from nerve terminals when its C-terminus is fully removed^20^ and by a recent report that Ca_V_2.1 channels with the C-terminus of Ca_V_2.3 exhibit impaired presynaptic abundance.^48^

Sequences outside the Ca_V_ C-terminus could also be involved. For example, binding of the synprint motif in the Ca_V_2 cytoplasmic II-III loop to SNARE proteins may contribute to release site recruitment.^49-51^ However, channels lacking this sequence retain presynaptic targeting, and synprint inhibitory peptides can act independently of the synprint motif.^52,53^ Interactions with material in the synaptic cleft may also mediate active zone anchoring.^54,55^ Taken together, multiple interactions have been implicated in Ca_V_ trafficking and targeting, but how these interactions direct Ca_V_1s and Ca_V_2s to opposing compartments has remained unclear.

Here, we found that the Ca_V_ C-termini are the primary determinants of compartment targeting in hippocampal neurons. Swapping the Ca_V_2.1 C-terminus onto Ca_V_1.3 targets the channel to the presynaptic active zone in Ca_V_2 knockout neurons. This chimeric Ca_V_1.3 channel mediates Ca^2+^ entry for neurotransmitter release and renders synaptic vesicle exocytosis sensitive to L-type Ca_V_ blockers. In contrast, the reverse swap prevents active zone localization and function of Ca_V_2.1. Within the Ca_V_2.1 proximal C-terminus, the first EF hand is required for presynaptic targeting, and its removal leads to loss of Ca_V_2.1 from the active zone. We conclude that the C-terminus specifies Ca_V_ localization, and we identify the first EF hand as an essential trafficking motif.

## RESULTS

### Exogenous Ca_V_2.1, but not Ca_V_1.3, localizes to active zones and mediates neurotransmitter release after Ca_V_2 ablation

**Figure 1.**
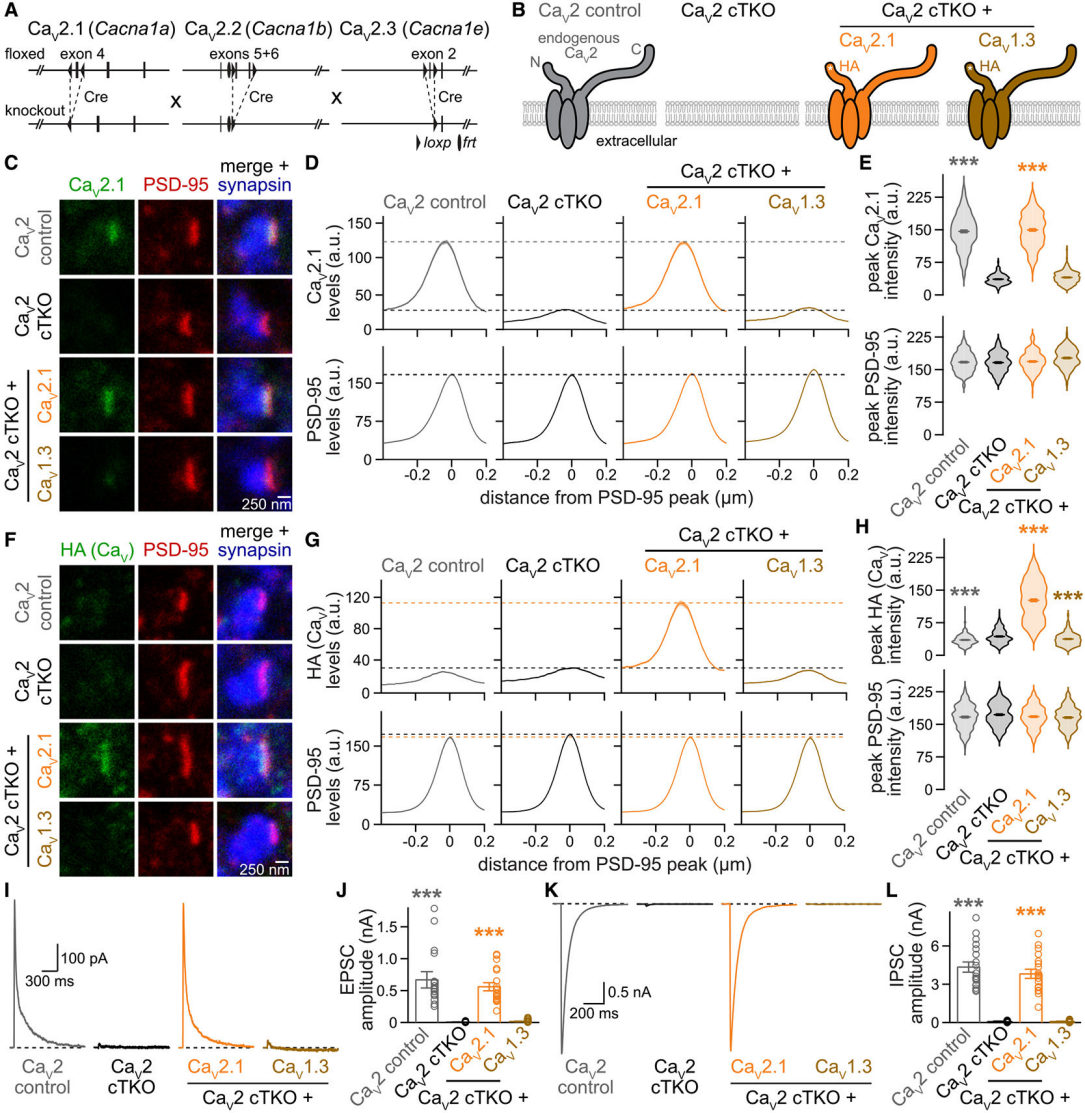
Lentivirally expressed Ca_V_2.1, but not Ca_V_1.3, localizes to active zones and restores synaptic transmission in Ca_V_2 triple knockout neurons (A) Strategy for Ca_V_2 triple knockout in cultured hippocampal neurons as described before.^20^ Transduction of neurons from triple-floxed mice with a lentivirus expressing Cre recombinase produced Ca_V_2 cTKO neurons. Ca_V_2 control neurons were identical except for the expression of a truncated, recombination-deficient Cre. (B) Schematic of the conditions for comparison (schematics similar to that in Held et al.^20^); HA-tagged (HA) Ca_V_s were expressed by lentiviral transduction. (C–E) Representative images (C) and summary plots of intensity profiles (D) and peak levels (E) of Ca_V_2.1 and PSD-95 at side-view synapses; levels are shown in arbitrary units (a.u.). Neurons were stained with antibodies against Ca_V_2.1 (analyzed by STED microscopy), PSD-95 (STED), and synapsin (confocal). Dashed lines in (D) denote levels in Ca_V_2 cTKO (black) and Ca_V_2 control (gray); Ca_V_2 control, 195 synapses/3 cultures; Ca_V_2 cTKO, 202/3; Ca_V_2 cTKO + Ca_V_2.1, 205/3; Ca_V_2 cTKO + Ca_V_1.3, 201/3. (F–H) As in (C)–(E) but for synapses stained with antibodies against HA (to detect lentivirally expressed Ca_V_s, STED), PSD-95 (STED), and synapsin (confocal). Dashed lines in (G) denote levels in Ca_V_2 cTKO (black) and Ca_V_2 cTKO + Ca_V_2.1 (orange); Ca_V_2 control, 208/3; Ca_V_2 cTKO, 222/3; Ca_V_2 cTKO + Ca_V_2.1, 227/3; Ca_V_2 cTKO + Ca_V_1.3, 214/3. (I and J) Representative traces (I) and quantification (J) of NMDAR-mediated EPSCs recorded in whole-cell configuration and evoked by focal electrical stimulation; 18 cells/3 cultures each. (K and L) As in (I) and (J) but for IPSCs; 18/3 each. Data are mean ± SEM; ***p < 0.001. Statistical significance compared to Ca_V_2 cTKO was determined with Kruskal-Wallis tests followed by Dunn’s multiple comparisons post hoc tests for the proteins of interest or amplitudes in (E), (H), (J), and (L). In (H), the small decreases in HA intensity in Ca_V_2 control and Ca_V_2 cTKO + Ca_V_1.3 compared to Ca_V_2 cTKO (which does not express an HA-tagged protein) are unlikely biologically meaningful. For Ca_V_ C-terminal sequences and additional Ca_V_ expression analyses, see Figure S1.

To determine the Ca_V_ sequences important for active zone localization, we expressed various Ca_V_s using lentiviruses in cultured hippocampal neurons that lack Ca_V_2.1, Ca_V_2.2, and Ca_V_2.3. Specifically, we transduced neurons that contain “floxed” conditional knockout alleles for these three channels (Figure 1A) with lentiviruses that express cre recombinase under a synapsin promoter to generate Ca_V_2 cTKO neurons.^20^ Control neurons (Ca_V_2 control) were identical except for transduction by a lentivirus expressing a truncated, recombination-deficient version of cre. In addition, we transduced Ca_V_2 cTKO neurons with a lentivirus expressing either hemagglutinin (HA)-tagged Ca_V_2.1 or HA-tagged Ca_V_1.3. The HA tag was inserted near the Ca_V_ N-terminus in a position shown previously to not interfere with the expression (Figures 1B and S1A-1E), targeting, and function of Ca_V_2.1.^20,56^ We then used stimulated emission depletion (STED) microscopy (Figures 1C-1H), confocal microscopy (Figures S1F-S1I), and electrophysiology (Figures 1I-1L) to assess Ca_V_ localization and synaptic transmission.

For morphological analyses, neurons were stained with antibodies against Ca_V_2.1 or HA to detect Ca_V_s, PSD-95 to mark postsynaptic densities, and synapsin to label synaptic vesicle clusters. For STED analyses (Figures 1C-1H), we selected synapses in side view through the presence of a vesicle cloud (imaged with confocal microscopy) and an elongated PSD-95 structure (imaged with STED microscopy) at one edge of the vesicle cloud, as established previously.^20,25,40,41,57^ We assessed Ca_V_ distribution and levels in STED images of these side-view synapses using line profiles perpendicular to PSD-95, and we plotted the average profiles (Figures 1D and 1G) and peak intensities (Figures 1E and 1H).

Endogenous and re-expressed Ca_V_2.1 formed elongated structures apposed to PSD-95 with a maximal intensity within tens of nanometers of the PSD-95 peak (Figures 1C-1H). We have established before that this distribution is characteristic of active zone localization.^20,25,41,58^ Furthermore, a strong PSD-95 peak was present in all conditions, matching our previous work that did not find morphological defects following Ca_V_2 triple knockout.^20^ Exogenously expressed Ca_V_1.3, monitored via the HA tag, was not detected at the active zone (Figures 1F-1H). Consistent with the STED analyses, confocal microscopy revealed robust levels of Ca_V_2.1, but not Ca_V_1.3, in synaptic regions of interest (ROIs) defined by synapsin (Figures S1F-S1I). Independent of their synaptic targeting, both Ca_V_2.1 and Ca_V_1.3 were effectively expressed in the somata of transduced Ca_V_2 cTKO neurons and in transfected HEK293T cells (Figures S1C-S1E).

These morphological experiments were complemented with analyses of synaptic transmission (Figures 1I-1L). A focal stimulation electrode was used to evoke action potentials, and inhibitory or excitatory postsynaptic currents (IPSCs or EPSCs, respectively) were isolated pharmacologically. EPSCs were monitored via NMDA receptors because network excitation confounds the interpretation of EPSC amplitudes when AMPA receptors are not blocked. Ca_V_2 cTKO nearly abolished evoked synaptic transmission, as characterized in detail before.^20^ Re-expression of Ca_V_2.1 restored EPSCs and IPSCs effectively, but exogenous expression of Ca_V_1.3 failed to produce any recovery (Figures 1I-1L), in agreement with the absence of Ca_V_1.3 from presynaptic sites (Figures 1F-1H, S1H, and S1I). Taken together, these results establish that exogenously expressed Ca_V_2.1, but not Ca_V_1.3, localizes to the active zone and gates neurotransmitter release in Ca_V_2 cTKO neurons.

### Ca_V_1.3 chimeras that contain the Ca_V_2.1 C-terminus localize to the active zone

**Figure 2.**
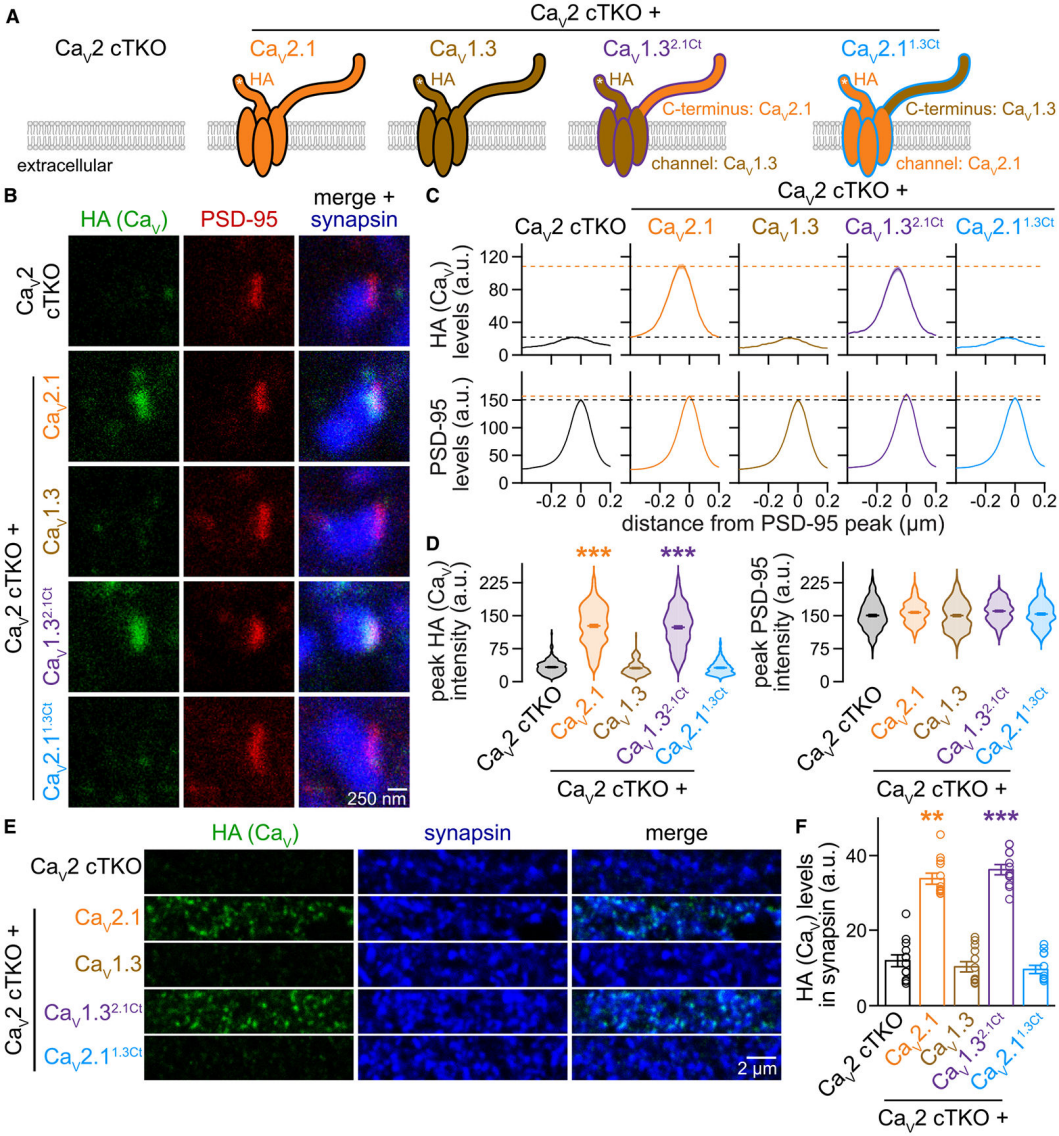
The Ca_V_2.1 C-terminus suffices to target Ca_V_1.3 to the presynaptic active zone (A) Schematic of the conditions for comparison. (B–D) Representative images (B) and summary plots of intensity profiles (C) and peak levels (D) of HA and PSD-95 at side-view synapses stained for HA (STED), PSD-95 (STED), and synapsin (confocal). Dashed lines in (C) denote levels in Ca_V_2 cTKO (black) and Ca_V_2 cTKO + Ca_V_2.1 (orange); Ca_V_2 cTKO, 205 synapses/3 cultures; Ca_V_2 cTKO + Ca_V_2.1, 203/3; Ca_V_2 cTKO + Ca_V_1.3, 222/3; Ca_V_2 cTKO + Ca_v_1.3^2.1Ct^, 218/3; Ca_V_2 cTKO + Ca_V_2.1^1.3Ct^, 208/3. (E and F) Representative areas of confocal images (E) and quantification (F) of HA levels in synapsin regions of interest (ROIs). Identical areas (58.14 × 58.14 μm^2^) were imaged for confocal (E) and (F) and STED (B)–(D) analyses, and whole images were quantified in (E) and (F); 12 images/3 cultures each. Data are mean ± SEM; **p < 0.01 and ***p < 0.001. Statistical significance compared to Ca_V_2 cTKO was determined with Kruskal-Wallis tests followed by Dunn’s multiple comparisons post hoc tests for the protein of interest in (D) and (F). For additional Ca_V_ expression analyses, see Figure S2.

Given the diverse interactions that converge within the Ca_V_ C-termini (Figures S1A and S1B),^20,44,45^ we hypothesized that the C-terminal sequences contain sufficient information to instruct Ca_V_ compartment specificity. To test this hypothesis, we generated two chimeric Ca_V_s. In Ca_V_1.3, we replaced the intracellular C-terminus immediately after the last transmembrane segment with that of Ca_V_2.1, generating a channel we named Ca_V_1.3^2.1Ct^. We also produced the inverse construct by replacing the Ca_V_2.1 C-terminus with that of Ca_V_1.3, generating Ca_V_2.1^1.3Ct^ (Figures 2A and S1A). Both chimeric channels were efficiently expressed in transfected HEK293T cells and robustly detected in neuronal somata following lentiviral transduction of Ca_V_2 cTKO neurons (Figure S2).

We then assessed the localization of these chimeric channels in the experimental setup described above and compared them side by side with Ca_V_2.1 and Ca_V_1.3. Strikingly, translocating the Ca_V_2.1 C-terminus onto Ca_V_1.3 efficiently targeted the resulting chimeric Ca_V_1.3^2.1Ct^ channel to the active zone in Ca_V_2 cTKO neurons, as assessed with STED microscopy (Figures 2B-2D). The distribution profile of Ca_V_1.3^2.1Ct^ and its abundance at the active zone recapitulated those of re-expressed Ca_V_2.1 (Figures 2B-2D). In contrast, the inverse swap abolished active zone localization of Ca_V_2.1^1.3Ct^ (Figures 2B-2D) despite effective somatic expression (Figures S2B and S2C). Confocal microscopic analyses of Ca_V_ levels in synaptic ROIs corroborated these findings by revealing robust synaptic localization of Ca_V_1.3^2.1Ct^ but not Ca_V_2.1^1.3Ct^ (Figures 2E and 2F).

These results establish that Ca_V_1.3 is targeted to the presynaptic active zone when its C-terminus is replaced with that of Ca_V_2.1. Conversely, Ca_V_2.1 loses its active zone localization following the reverse swap. We conclude that the Ca_V_ C-termini contain sufficient information to define Ca_V_ compartment specificity, and these and previous data lead to two predictions. First, because removing known scaffolding motifs in the distal C-terminus only partially impaired active zone localization,^20,47^ there must be essential targeting motifs in the Ca_V_ C-terminus that have not yet been identified. Second, if the chimeric Ca_V_1.3^2.1Ct^ channel is appropriately coupled to primed vesicles within the active zone, then Ca_V_1.3^2.1Ct^ expression should restore synaptic transmission in Ca_V_2 cTKO neurons and render neurotransmitter release sensitive to L-type channel blockade. We next tested both predictions.

### The first EF hand in the proximal C-terminus is necessary for Ca_V_2 active zone targeting

**Figure 3.**
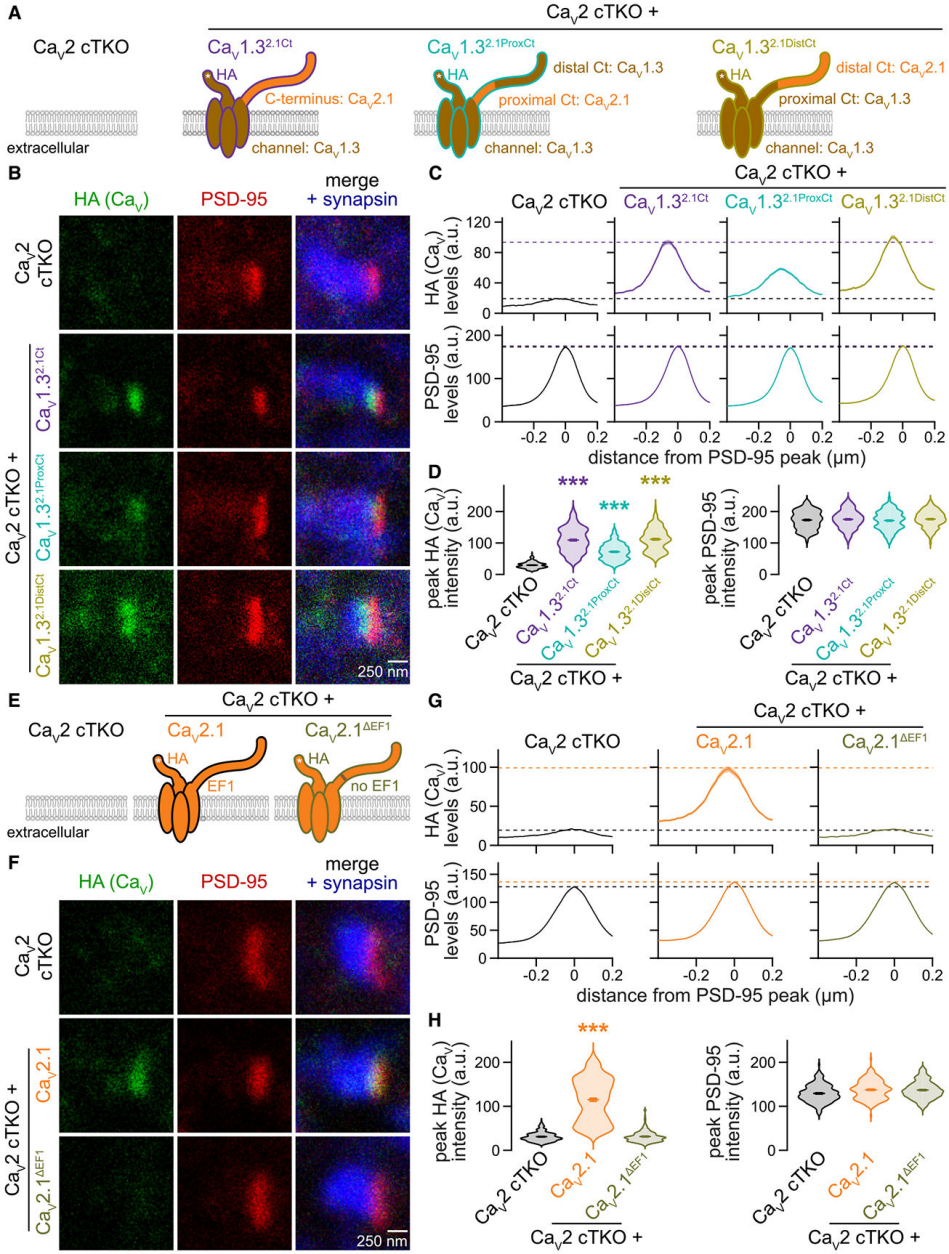
The first EF hand in the proximal C-terminus is essential for Ca_V_2 active zone targeting (A) Schematic of the conditions for comparison in (B)–(D). (B–D) Representative images (B) and summary plots of intensity profiles (C) and peak levels (D) of HA and PSD-95 at side-view synapses stained for HA (STED), PSD-95 (STED), and synapsin (confocal). Dashed lines in (C) denote levels in Ca_V_2 cTKO (black) and Ca_V_2 cTKO + Ca_V_1.3^2.1Ct^ (purple); Ca_V_2 cTKO, 207 synapses/3 cultures; Ca_V_2 cTKO + Ca_V_1.3^2.1Ct^, 204/3; Ca_V_2 cTKO + Ca_V_1.3^2.1ProxCt^, 209/3; Ca_V_2 cTKO + Ca_V_1.3^2.1DistCt^, 210/3. (E) Schematic of the conditions for comparison in (F)–(H). (F–H) Representative images (F) and summary plots of intensity profiles (G) and peak levels (H) of HA and PSD-95 at side-view synapses stained for HA (STED), PSD-95 (STED), and synapsin (confocal). Dashed lines in (G) denote levels in Ca_V_2 cTKO (black) and Ca_V_2 cTKO + Ca_V_2.1 (orange); Ca_V_2 cTKO, 200/3; Ca_V_2 cTKO + Ca_V_2.1, 180/3; Ca_V_2 cTKO + Ca_V_2.1^ΔEF1^, 203/3. Data are mean ± SEM; ***p < 0.001. Statistical significance compared to Ca_V_2 cTKO was determined with Kruskal-Wallis tests followed by Dunn’s multiple comparisons post hoc tests for the protein of interest in (D) and (H). For additional Ca_V_ expression analyses, see Figure S3.

Removal of the known active zone scaffolding motifs in the Ca_V_2.1 C-terminus produced a partial defect in Ca_V_2.1 active zone targeting, but truncation of the entire C-terminus fully abolished active zone localization.^20^ To define C-terminal sequences that contain unidentified targeting motifs, we segregated the Ca_V_2.1 C-terminus into a distal segment containing the active zone scaffolding motifs and a complementary proximal segment (Figures S1A and S1B). We generated two additional Ca_V_1.3 chimeras (Figure 3A) by translocating either only the Ca_V_2.1 proximal (Ca_V_1.3^2.1ProxCt^) or distal (Ca_V_1.3^2.1DistCt^) C-terminus onto Ca_V_1.3. Ca_V_1.3^2.1ProxCt^ and Ca_V_1.3^2.1DistCt^ were expressed efficiently in HEK293T cells after transfection and in neuronal somata after lentiviral transduction (Figures S3A-S3C). With STED microscopy, we detected Ca_V_1.3^2.1ProxCt^ at the active zone (Figures 3B-3D) of Ca_V_2 cTKO neurons. Active zone levels of Ca_V_1.3^2.1ProxCt^ were reduced compared to Ca_V_1.3^2.1Ct^ levels and resembled those of a mutant Ca_V_2.1 that lacks the active zone scaffolding motifs in the distal C-terminus.^20^ Hence, active zone targeting of chimeric Ca_V_1.3s operates in part through these distal sequences. Accordingly, Ca_V_1.3^2.1DistCt^ exhibited strong active zone localization in Ca_V_2 cTKO neurons and was indistinguishable from Ca_V_1.3^2.1Ct^ (Figures 3B-3D). Confocal analyses of protein levels in synaptic ROIs matched these findings (Figures S3D and S3E).

Ca_V_1.3^2.1ProxCt^ demonstrates that translocation of the Ca_V_2.1 proximal C-terminus onto Ca_V_1.3 suffices to mediate some active zone localization (Figures 3B-3D) and indicates that the proximal C-terminal sequences are important for presynaptic trafficking. Ca_V_ proximal C-termini (Figures S1A and S1B) contain two EF hands.^28,59^ The first EF hand has been implicated in calmodulin-dependent modulation of Ca_V_ function,^60-62^ though no evidence to date establishes a role in Ca_V_ trafficking. We tested whether the first EF hand mediates active zone targeting by deleting it from Ca_V_2.1 (Ca_V_2.1^ΔEF1^; Figure 3E). Ca_V_2.1^ΔEF1^ was readily expressed in transfected HEK293T cells and detected in somata of lentivirally transduced neurons (Figures S3F-S3H). However, deleting this EF hand abolished Ca_V_2.1 active zone localization in STED microscopy (Figures 3F-3H) and rendered Ca_V_2.1^ΔEF1^ undetectable at synapses in confocal microscopy (Figures S3I and S3J).

In summary, the Ca_V_2.1 distal C-terminus needs to be paired with proximal C-terminal elements to effectively localize Ca_V_s to the active zone. Our data establish that the first EF hand is required for active zone targeting of Ca_V_2.1.

### Ca_V_1.3^2.1Ct^ supports neurotransmitter release and confers L-type blocker sensitivity after Ca_V_2 ablation

**Figure 4.**
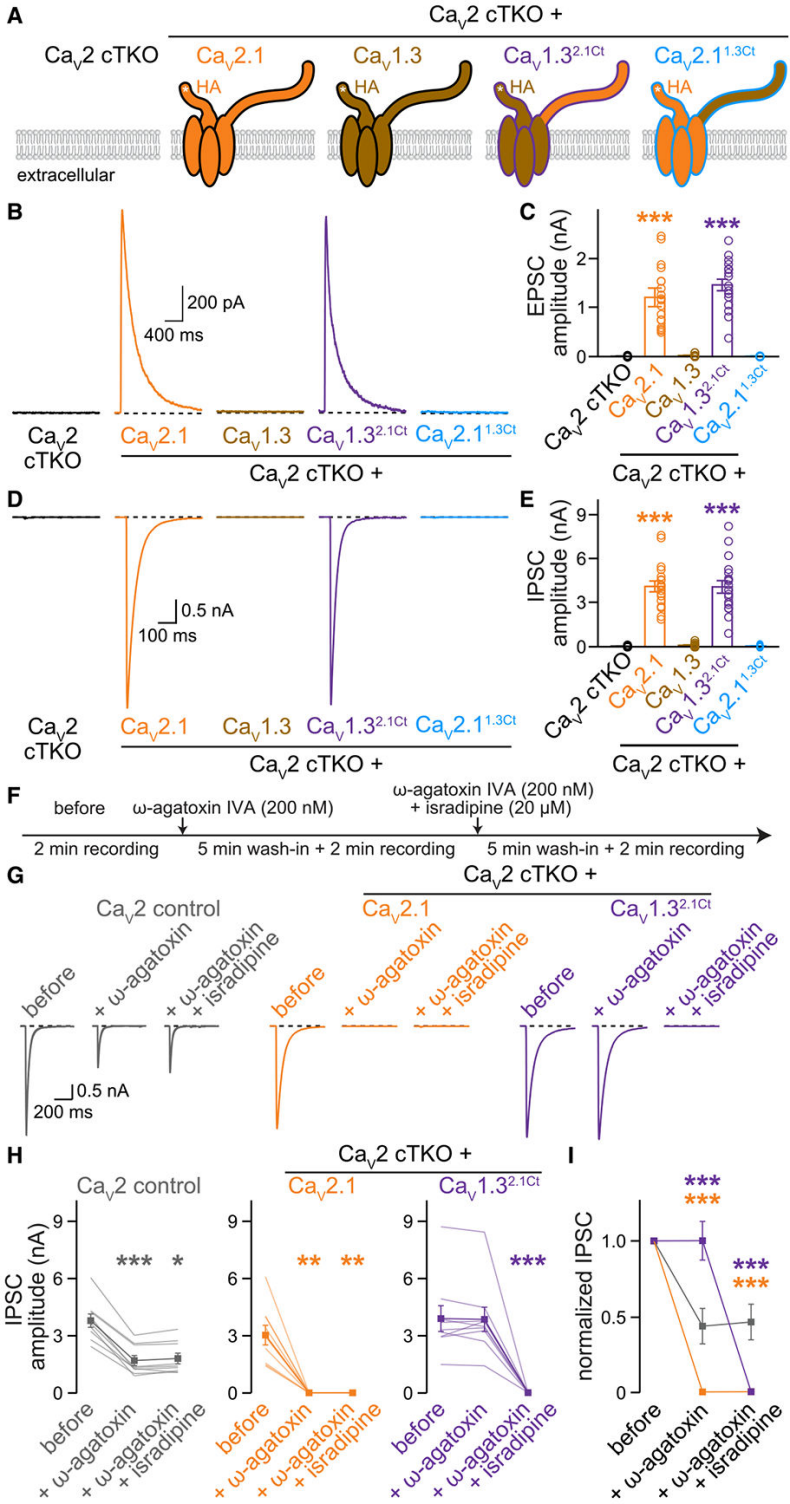
Ca_V_1.3^2.1Ct^ channels mediate neuro-transmitter release and render it L-type blocker sensitive (A) Schematic of the conditions for comparison. (B and C) Representative traces (B) and quantification (C) of NMDAR-mediated EPSCs; 18 cells/3 cultures each. (D and E) As in (B) and (C) but for IPSCs; 18/3 each. (F) Experimental strategy to evaluate blocker sensitivity of synaptic transmission. Evoked IPSCs were recorded before blocker application (before), after wash-in of 200 nM ω-agatoxin IVA alone (+ ω-agatoxin, to block Ca_V_2.1), and after wash-in of 200 nM ω-agatoxin IVA and 20 μM isradipine (+ ω-agatoxin + isradipine, to block Ca_V_1s and Ca_V_2.1). (G and H) Representative traces (G) and quantification (H) of IPSCs recorded as outlined in (F); 9/3 each. (I) Comparison of IPSCs normalized to “before” in each condition; 9/3 each. Data are mean ± SEM; *p < 0.05, **p < 0.01, and ***p < 0.001. Statistical significance compared to Ca_V_2 cTKO was determined with Kruskal-Wallis tests followed by Dunn’s multiple comparisons post hoc tests in (C) and (E). Statistical significance compared to “before” was determined with Friedman tests followed by Dunn’s multiple comparisons post hoc tests in (H). Statistical significance compared to Ca_V_2 control was determined with two-way, repeated-measures ANOVA followed by Dunnett’s multiple comparisons post hoc tests in (I). For additional electrophysiological data, see Figure S4. For characterization of C-terminally truncated Ca_V_1.3, see Figure S5.

Having demonstrated that translocation of the Ca_V_2.1 C-terminus directs Ca_V_1.3 to the active zone, we asked whether Ca_V_1.3^2.1Ct^ provides Ca^2+^ for triggering synaptic vesicle exocytosis (Figure 4A). We first evaluated Ca_V_1.3^2.1Ct^ by characterizing its activity in transfected HEK293T cells. Ca_V_1.3^2.1Ct^ conducted robust inward currents, and Ca_V_1.3^2.1Ct^ and Ca_V_1.3 exhibited similar voltage dependency (Figures S4A-S4C). When expressed in Ca_V_2 cTKO neurons, Ca_V_1.3^2.1Ct^ restored evoked EPSCs (Figures 4B and 4C) and IPSCs (Figures 4D and 4E) as well as decreases in miniature EPSC (mEPSC) and mIPSC frequencies,^20^ as did Ca_V_2.1 (Figures S4D-S4G). These data establish that Ca_V_1.3^2.1Ct^ is coupled to the presynaptic release machinery to gate neurotransmitter release. In contrast, and consistent with the loss of active zone targeting in neurons (Figures 2B-2D) and the inability to mediate inward currents in HEK293T cells (Figures S4A-S4C), Ca_V_2.1^1.3Ct^ failed to restore evoked synaptic transmission (Figures 4B-4E).

It is possible that Ca_V_1.3^2.1Ct^ presynaptic targeting and function result from the removal of a dendritic targeting sequence rather than the addition of an axonal targeting motif. To address this possibility, we generated a Ca_V_1.3 lacking the entire C-terminus (Ca_V_1.3^ΔCt^). Ca_V_1.3^ΔCt^ was effectively expressed (Figures S5A-S5D) but not targeted to synapses (Figures S5E and S5F) or active zones (Figures S5G-S5I). Furthermore, Ca_V_1.3^ΔCt^ did not mediate neurotransmitter release (Figures S5J-S5M). We conclude that active zone targeting of Ca_V_1.3^2.1Ct^ arises from an instructive role of the Ca_V_2.1 C-terminus.

At central synapses, neurotransmitter release is insensitive to L-type Ca_V_ blockade (Figures S4H-S4J).^17^ Given that we replaced presynaptic Ca_V_2s with the L-type-like Ca_V_1.3^2.1Ct^, we tested whether we also altered the pharmacological sensitivity of synaptic transmission. We performed serial Ca_V_ blockade (Figure 4F) through sequential application of ω-agatoxin IVA (ω-agatoxin, to block Ca_V_2.1) and isradipine (to block Ca_V_1s). In Ca_V_2 control neurons, ω-agatoxin reduced IPSCs by half (Figures 4G-4I), consistent with the reliance of these synapses on both Ca_V_2.1 and Ca_V_2.2.^20,24,63^ Isradipine had no effect in Ca_V_2 control neurons (Figures S4H-S4J). Naturally, ω-agatoxin fully inhibited synaptic transmission in Ca_V_2 cTKO neurons rescued with Ca_V_2.1. However, for Ca_V_2 cTKO neurons that expressed Ca_V_1.3^2.1Ct^, synaptic transmission was resistant to ω-agatoxin and instead wholly sensitive to isradipine (Figures 4G-4I). Hence, Ca_V_1.3^2.1Ct^ functionally incorporates into release machinery in Ca_V_2 cTKO neurons and renders neurotransmission fully dependent on L-type Ca_V_ activity.

## DISCUSSION

Distinct Ca_V_s are sorted effectively to somatodendritic and axonal compartments. Here, we establish that the Ca_V_ C-termini contain necessary and sufficient information for compartment sorting. Within the C-terminus of Ca_V_2.1, the first EF hand is essential for presynaptic targeting, and it operates in concert with distal scaffolding motifs. Together, the Ca_V_2.1 C-terminal sequences are sufficient to re-direct somatodendritic Ca_V_1 channels to the active zone of axonal nerve terminals. Conversely, the Ca_V_1.3 C-terminal sequences disrupt Ca_V_2.1 active zone localization and its function. Our work establishes mechanisms for compartment-specific targeting of a protein family central to the polarized organization of neurons.

### Forward trafficking of Ca_V_s

Multiple cargo selectivity filters within the endoplasmic reticulum, Golgi apparatus, axon initial segment, and presynaptic boutons together permit the targeting of a limited subset of proteins to the active zone while deflecting other cargo.^64,65^ Sequence motifs within these proteins may dictate compartment sorting at two major checkpoints: (1) they may mediate protein recruitment into cargo vesicles directed to the axon, and (2) they may stabilize proteins at the active zone following their delivery.^2,66^ Our work establishes that the Ca_V_2.1 C-terminus encodes necessary and sufficient information to navigate these check-points and implies a cooperative relationship between the proximal and distal elements. The Ca_V_2.1 distal C-terminus efficiently localizes chimeric Ca_V_1.3s to the active zone, indicating that the distal sequences permit both Ca_V_ sorting into presynaptic cargo and Ca_V_ tethering at the active zone, so long as the first EF hand is present. The distal motifs that bind to active zone proteins likely fulfill these roles, as disrupting their interactions with RIM and RIM-BP leads to targeting defects,^20,24,38,39,41,47^ similar to those exhibited by chimeric Ca_V_1.3s with the Ca_V_2.1 proximal C-terminus and the Ca_V_1.3 distal C-terminus (Figure 3).

Both Ca_V_1.3^2.1Ct^ and Ca_V_1.3^2.1DistCt^ are efficiently targeted to the active zone, which establishes that the Ca_V_1.3 and Ca_V_2.1 proximal C-termini contain information for Ca_V_ active zone delivery when paired with the Ca_V_2.1 distal scaffolding motifs. This is in line with the high homology of the EF hands and IQ motif across Ca_V_ proximal C-termini and the presence of these sequences in other voltage-gated channels.^28,67^ The proximal C-terminus might provide multiple instructive signals. For example, the first EF hand binds AP-1, and the IQ motif binds calmodulin, and these interactions could serve as trafficking control checkpoints.^33,34,68,69^ Proximal C-terminal sequences are also involved in Ca^2+^-dependent signaling,^60-62^ but any contributions to trafficking are likely independent of these functions as perturbations of Ca^2+^ influx and neuronal firing do not disrupt presynaptic Ca_V_ abundance.^20^ Unknown interactions of the proximal C-terminus may be involved in targeting as well. Altogether, we posit that the first EF hand is necessary for the incorporation of Ca_V_s into axon-bound cargo, but it likely has no role afterward in stabilizing Ca_V_s within the active zone.

### C-terminal sequences for regulating Ca_V_ abundance at the active zone

Ca_V_2 active zone targeting may be regulated by the availability of channel slots, which may impose an upper limit on presynaptic Ca_V_ abundance and may prefer specific Ca_V_ subtypes.^70,71^ We found that Ca_V_2.1 and Ca_V_1.3^2.1Ct^ restored active zone Ca_V_ abundance and neurotransmitter release with similar efficacy when expressed in Ca_V_2 cTKO neurons. Importantly, synaptic transmission in these neurons and in Ca_V_2 control neurons was comparable (Figures 2, 4, and S4). Hence, Ca_V_s with the Ca_V_2.1 C-terminus effectively occupy available Ca_V_ slots in terminals without endogenous Ca_V_2s. In contrast, recent work revealed that swapping the Ca_V_2.3 C-terminus onto Ca_V_2.1 decreased channel abundance at the calyx of Held,^48^ suggesting that the Ca_V_2.3 C-terminus does not efficiently target channels to the same slots. Overall, the Ca_V_2.1 C-terminus mediates efficient slot occupancy, and Ca_V_2 C-termini may also define slot preference.

Ca_V_2 alternative splicing may confer further diversity. Ca_V_2 C-termini contain multiple splice sites that may regulate channel trafficking.^72,73^ For example, alternative splicing can remove the distal scaffolding motifs necessary for efficient active zone tethering.^20,72^ In Ca_V_2.2s, splicing within the first EF hand can disrupt an AP-1 binding site, possibly impeding forward trafficking.^68^ In summary, Ca_V_s undergo extensive alternative splicing, but how splicing modulates Ca_V_ localization and function remains unclear.

### Limitations of the study

Because we characterized Ca_V_2.1, Ca_V_1.3, and their derivative chimeric channels in Ca_V_2 cTKO neurons, we did not assess the relative contributions of endogenous Ca_V_2.1, Ca_V_2.2, and Ca_V_2.3 to evoked and spontaneous neurotransmitter release or the possibility of subtype-preferring slots.^17-20,70,71,74-79^ Ca_V_2 channels can exhibit some somatodendritic activity and localization.^80-83^ The underlying targeting mechanisms are unknown, and it remains uncertain whether this localization arises from active somatodendritic retention. Further, we utilize virally expressed Ca_V_ cDNAs for rescue, which prevents alternative splicing that may further modulate channel abundance, localization, and function.^61,68,72,73,84^ Finally, the cultured neurons were mixed from neonatal mice of both sexes and cannot be used to assess sex-specific phenotypes.

Nevertheless, our work provides mechanistic insight into the polarized trafficking of protein material in neurons and raises multiple questions. First, some synapses depend on only a single Ca_V_2 subtype, while others use multiple Ca_V_2s, and some synapses experience developmental switches in Ca_V_2 usage.^85,86^ Whether there are specific trafficking mechanisms or whether these properties are determined wholly by switches in gene expression remains to be determined. Second, the proximal sequences we identified as important for targeting are also present in other ion channels that undergo polarized trafficking, for example in Na^+^ channels.^28,67^ It is possible that the mechanisms we describe for Ca_V_s are employed by other channels, and Ca_V_s represent an ideal framework to further define mechanisms that sort proteins into specific neuronal compartments.

## STAR★METHODS

### RESOURCE AVAILABILITY

#### Lead contact

Further information and requests for resources and reagents should be directed to and will be fulfilled by the lead contact, Pascal S. Kaeser (kaeser@hms.harvard.edu).

#### Materials availability

Plasmids generated for this study will be shared upon request. Mouse lines and previously generated plasmids will be shared upon request within the limits of the respective material transfer agreements.

#### Data and code availability

- Data reported in this paper will be shared by the lead contact upon request. A table with numerical data has been deposited at Zenodo and is available at https://doi.org/10.5281/zenodo.11583935.
- This paper does not report original code.
- Any additional information required to reanalyze the data reported in this paper is available from the lead contact upon request.

### EXPERIMENTAL MODEL AND SUBJECT DETAILS

#### Mice

Ca_V_2 conditional triple homozygote floxed mice were described before^20^ and they contain homozygote floxed alleles for Ca_V_2.1 (*Cacna1a*,^87^), Ca_V_2.2 (*Cacna1b*,^20^), and Ca_V_2.3 (*Cacna1e*,^88^). Adult mice were either separated by sex, or housed as breeding pairs, and they were under a 12 h light-dark cycle with free access to food and water in a room set to 22°C (range 20°C–24°C) and 50% humidity (range 35–70%). Mice were genotyped either in the lab following established protocols^20^ or by Transnetyx. For *Cacna1a*, the following oligonucleotide primer pair was used for in-lab genotyping: forward, ACCTACAGTCTGCCAGGAG; reverse, TGAAGCCCAGACATCCTTGG (expected band sizes, wild type: 393 bp, floxed: 543 bp); for *Cacna1b*: forward, TGGTTGG TGTCCTGTTCTCC; reverse, TAAGGAGCAGGGAATCCTGG (expected band sizes, wild type: 219bp, floxed: 359 bp); for *Cacna1c*: forward, GACAAGACCCCAATGTCTCG; reverse, TCCATGTTCCTTCTCACTCC (expected band sizes, wild type: 295 bp, floxed: 334 bp). Animal experiments were performed according to approved protocols at Harvard University.

#### Primary neuronal cultures

Primary mouse hippocampal cultures were generated from newborn mice as established before.^20,40,41^ Hippocampi were dissected out from newborn mice within 24 h after birth. Cells were dissociated and plated onto Matrigel-treated glass coverslips in plating medium composed of Minimum Essential Medium (MEM) with 0.5% glucose, 0.02% NaHCO3, 0.1 mg/mL transferrin, 10% Fetal Select bovine serum (Atlas Biologicals FS-0500-AD), 2 mM L-glutamine, and 25 μg/mL insulin. Cells from mice of both sexes were mixed. Cultures were maintained in a 37°C-tissue culture incubator, and after ~24 h the plating medium was exchanged with growth medium composed of MEM with 0.5% glucose, 0.02% NaHCO3, 0.1 mg/mL transferrin, 5% Fetal Select bovine serum (Atlas Biologicals FS-0500-AD), 2% B-27 supplement (Thermo Fisher 17504044), and 0.5 mM L-glutamine. On day *in vitro* (DIV) 1 to 2, depending on growth, 50% or 75% of the medium was exchanged with growth medium supplemented with 4 μM Cytosine β-D-arabinofurano-side (AraC). Experiments and analyses were performed at DIV15 to 19.

#### Cell lines

HEK293T cells, an immortalized cell line of female origin, were cultured as established before.^20,40,41^ They were purchased from ATCC (CRL-3216, RRID: CVCL_0063), expanded, and stored in liquid nitrogen until use. After thawing, the cells were grown in Dulbecco’s Modified Eagle Medium with 10% fetal bovine serum (Atlas Biologicals F-0500-D) and 1% penicillin-streptomycin. HEK293T cells were passaged every 1 to 3 d at a ratio of 1:3 to 1:10. HEK293T cell batches were typically replaced after 20 passages by thawing a fresh vial from the expanded stock.

### METHOD DETAILS

#### Lentiviruses

Lentiviruses used to transduce primary hippocampal neurons were produced in HEK293T cells. HEK293T cells were transfected with the Ca^2+^ phosphate method with REV (p023), RRE (p024) and VSVG (p025), as well as a lentiviral plasmid encoding the protein of interest. For Ca_V_ proteins of interest, these were plasmids p789, p947, p1077, p1078, p1079, p1080, p1083, and p1084. To produce lentiviruses expressing EGFP-tagged Cre recombinase (to generate Ca_V_2 cTKO neurons), pFSW EGFP-Cre (p009) was used. For lentiviruses expressing a truncated, enzymatically inactive EGFP-tagged Cre (to generate Ca_V_2 control neurons), pFSW EGFP-ΔCre (p010) was used. Plasmids were transfected at a 1:1:1:1 molar ratio and with a total amount of 6.7 μg DNA. Approximately 24 h after transfection, the medium was switched to neuronal growth medium (described above), and the HEK293T cell supernatant was harvested 24–36 h later by centrifugation at 700 x g. For expression of EGFP-Cre and EGFP-ΔCre, neurons were transduced by adding HEK293T cell supernatant at DIV5. For expression of Ca_V_s, neurons were transduced at DIV1. Ca_V_2 control neurons were additionally transduced with a virus made using a pFSW plasmid (p008) lacking a cDNA in the multiple cloning site in place of an expression virus. Neuronal protein expression from these lentiviruses was driven by the human synapsin I promoter.^40,89^

#### Ca_V_ expression constructs

For experiments in neurons, lentiviral backbones containing the human synapsin I promoter were used (pFSW HA-Ca_V_2.1, p789; pFSW HA-Ca_V_2.1^ΔEF1^, p947; pFSW HA-Ca_V_1.3, p1077; pFSW HA-Ca_V_1.3^2.1Ct^, p1078; pFSW HA-Ca_V_2.1^1.3Ct^, p1079; pFSW HA-Ca_V_1.3^ΔCt^, p1080; pFSW HA-Ca_V_1.3^2.1ProxCt^, p1083; pFSW HA-Ca_V_1.3^2.1DistCt^, p1084). For experiments in HEK293T cells, expression vectors with a CMV promoter were used (pCMV HA-Ca_V_2.1, p771; pCMV HA-Ca_V_2.1^ΔEF1^, p939; pCMV HA-Ca_V_1.3, p1073; pCMV HA-Ca_V_1.3^2.1Ct^, p1074; pCMV HA-Ca_V_2.1^1.3Ct^, p1075; pCMV HA-Ca_V_1.3^μCt^, p1076; pCMV HA-Ca_V_1.3^2.1ProxCt^, p1081; pCMV HA-Ca_V_1.3^2.1DistCt^, p1082). For these constructs, the Ca_V_ coding sequences were identical between corresponding pFSW and pCMV versions. The sequence of Ca_V_2.1 was identical to GenBank Entry AY714490.1 (mouse) with the addition of an HA-tag after position V_27_ flanked by short, exogenous linkers. The resulting cDNAs (p771 and p789) had the sequence M_1_ARF … GVVV_27_-AS-YPYDVPDYA-ACR-G_28_AAG … DDWC_2369_. The sequence of Ca_V_1.3 was as follows: the pore region was identical to residues M_1_QHQ … FDYL_1466_ from Ca_V_1.3e[8a,11,31b,Δ32,42a] (rat) and corresponds to residues M_10_QHQ … FDYL_1475_ of GenBank Entry EDL89004.1. Ca_V_1.3e[8a,11,31b,Δ32,42a] was a gift from D. Lipscombe (Addgene Plasmid #49333; http://n2t.net/addgene:49333; RRID:Addgene_49333).^92^ The intracellular C-terminal tail was identical to residues T_7_ to L_695_ from GenBank Entry AF370010.1 (a partial cDNA, rat); a Ca_V_1.3 plasmid containing this C-terminal tail was a gift from I. Bezprozvanny.^45^ An HA-tag was inserted after position G_29_ (referring to the numbering of Addgene Plasmid #49333) and flanked by short, exogenous linkers. The resulting cDNAs (p1073 and p1077) had the sequence M_1_QHQ … SGEG_29_-AS-YPYDVPDYA-ACR-P_30_TSQ … FDYL_1466_-T_1467_RDW … ITTL_2155_, with M_1_QHQ-SGEG_29_ and P_30_TSQ-FDYL_1466_ derived from Addgene Plasmid #49333,^92^ and with T_1467_RDW-ITTL_2155_ derived from the plasmid obtained from I. Bezprozvanny.^45^ The sequence of Ca_V_^2.1Ct^ (p1074 and p1078) contained the pore region (MQHQ … DWSI) from p1077 (Ca_V_1.3) followed by the C-terminus (LGPH … DDWC) from p789 (Ca_V_2.1, see Figure S1B). The sequence of Ca_V_2.1^1.3Ct^ (p1075 and p1079) contained the pore region (MARF … FEYL) from p789 (Ca_V_2.1) followed by the C-terminus (TRDW … ITTL) from p1077 (Ca_V_1.3, see Figure S1B). The sequence of Ca_V_1.3^2.1ProxCt^ (p1081 and 1083) contained the pore region (MQHQ … DWSI) from p1077 (Ca_V_1.3), followed by the proximal C-terminus (LGPH … QAMR) from p789 (Ca_V_2.1) and then by the distal C-terminus (GKYP … ITTL) from p1077 (Ca_V_1.3, see Figure S1B). The sequence of Ca_V_1.3^2.1DistCt^ (p1082 and 1084) contained the pore region and the proximal C-terminus (MQHQ … QGLV) from p1077 (Ca_V_1.3) followed by the distal C-terminus (EEQN … DDWC) from p789 (Ca_V_2.1, see Figure S1B). In the sequence of Ca_V_2.1^ΔEF1^ (p939 and p947), the first EF hand (EYVR … LRVI) was replaced with residues EY in p789 (Ca_V_2.1, see Figure S1B). The sequence of Ca_V_1.3^ΔCt^ (p1076 and 1080) contained the pore region (MQHQ … DWSI) from p1077 (Ca_V_1.3) and did not contain a C-terminus (see Figure S1B).

#### Confocal and STED microscopy of synapses

Confocal and STED microscopy and analyses were performed as established before.^20,25,41,58,93,94^ Neurons cultured on 0.17 mm thick 12 mm diameter (#1.5) coverslips were washed two times with PBS warmed to 37°C, and then fixed in 2% PFA with 4% sucrose (in PBS) at room temperature. After fixation, coverslips were rinsed three times in PBS with 50 mM glycine, then permeabilized in PBS with 0.1% Triton X-100 and 3% BSA (TBP) for 1 h at room temperature. Coverslips were stained with primary antibodies diluted in TBP for ~48 h at 4°C. The following primary antibodies were used: mouse IgG1 anti-HA (1:500, RRID: AB_2565006, A12), rabbit anti-Ca_V_2.1 (1:200, RRID: AB_2619841, A46), guinea pig anti-PSD-95 (1:500, RRID: AB_2619800, A5), rabbit anti-synapsin (1:500, RRID: AB_2200097, A30), and mouse IgG1 anti-synapsin (1:500, RRID_2617071, A57). After primary antibody staining, coverslips were rinsed twice and washed three times for 5 min in PBS with 50 mM glycine at room temperature. Alexa Fluor 488 (to detect HA-tagged Ca_V_s or endogenous Ca_V_2.1; anti-mouse IgG1, RRID: AB_2535764, S7; or, anti-rabbit, RRID: AB_2576217, S5), Alexa Fluor 555 (to detect the postsynaptic marker PSD-95; anti-guinea pig, RRID: AB_2535856, S23), and Alexa Fluor 633 (to detect the synaptic vesicle cluster; anti-rabbit, RRID: AB_2535731, S33; or, anti-mouse IgG1, RRID: AB_2535768, S29) conjugated antibodies were diluted in TBP at 1:200 (for Alexa Fluor 488 and 555) or 1:500 (for Alexa Fluor 633), and coverslips were incubated with the secondary antibody solution for ~24 h at 4°C. Coverslips were then rinsed twice with PBS with 50 mM glycine and once with deionized water, air-dried and mounted on glass slides in fluorescent mounting medium. Confocal and STED images were acquired on a Leica SP8 Confocal/STED 3X microscope with a 100x oil-immersion 1.44 numerical aperture objective and gated detectors as established before.^20,93^ 58.14 x 58.14 μm^2^ areas were acquired using 2x digital zoom (4096 x 4096 pixel^2^, pixel size of 14.194 × 14.194 nm^2^). Alexa Fluor 633, Alexa Fluor 555, and Alexa Fluor 488 were excited at 633 nm, 555 nm and 488 nm using a white light laser at 1–10% of 1.5 mW laser power. The Alexa Fluor 633, Alexa Fluor 555, and Alexa Fluor 488 channels were acquired first in confocal mode. For the Alexa Fluor 555 and Alexa Fluor 488 channels, the same areas were then sequentially acquired in STED mode using 660 nm and 592 nm depletion lasers, respectively. Identical imaging and laser settings were applied to all conditions within a given biological repeat. For analyses of presynaptic Ca_V_ distribution in STED images, synapses were selected in side-view. Side-view synapses were defined as synapses that contained a synaptic vesicle cluster labeled with synapsin and were associated with an elongated PSD-95 structure along the edge of the vesicle cluster as established before.^20,41,57,93,95^ For intensity profile analyses, a ~1000 nm long, 200 nm wide, rectangular ROI was drawn perpendicular and across the center of the PSD-95 structure, and the intensity profiles were obtained using this ROI for both the protein of interest and PSD-95. To align individual profiles, the PSD-95 signal only was smoothened using a rolling average of 5 pixels, and the smoothened signal was used to define the peak position of PSD-95. The profiles for the protein of interest (Ca_V_ or HA) and smoothened PSD-95 were aligned to the PSD-95 peak position, averaged across synapses, and then plotted. Peak intensities were also analyzed by extracting the maximal value from the line profiles of the protein of interest (Ca_V_ or HA) and smoothened PSD-95 within a 200 nm window around the PSD-95 peak. Peak intensity values were plotted for each synapse and averaged. For quantification of confocal images, a custom MATLAB program (https://doi.org/10.5281/zenodo.6388196) was used to generate masks of the presynaptic marker (synapsin), with the threshold determined by automatic two-dimensional segmentation (Otsu algorithm).^91,95^ Regions of interest (ROIs) were defined as synapsin-positive areas formed by contiguous pixels of at least 0.05 μm^2^ in size. Each image typically contained between 500 and 1500 synapsin ROIs. Levels of HA or Ca_V_2.1 within these ROIs were measured and the average intensity across all ROIs within an image was calculated and plotted. Representative images in figures were cropped, rotated with bi-linear interpolation, and then brightness and contrast adjusted to facilitate inspection. Brightness and contrast adjustments were made for display in figures and were done identically for images within an experiment, but image quantification was performed on raw images without these adjustments. The experimenter was blind to the condition/genotype for image acquisition and analyses for STED and confocal microscopic experiments.

#### Confocal imaging of neuronal somata

Neurons cultured on 0.17 mm thick 12 mm diameter (#1.5) coverslips were washed with PBS warmed to 37°C and fixed in 2% PFA with 4% sucrose for 10 min at room temperature. Coverslips were then rinsed three times in PBS with 50 mM glycine at room temperature, permeabilized in TBP for 1 h at room temperature, and incubated in primary antibodies for ~48 h at 4°C. The following primary antibodies were used: mouse IgG1 anti-HA (1:500, RRID: AB_2565006, A12) and mouse IgG2b anti-NeuN (1:500, RRID: AB_10711040, A254). After staining with primary antibodies, coverslips were rinsed twice and washed three times for 5 min in PBS with 50 mM glycine at room temperature. Alexa Fluor 555 (to detect HA; anti-mouse IgG1, RRID: AB_2535769, S19), and 633 (to detect neuronal somata; anti-mouse IgG2b, RRID: AB_1500899, S31) conjugated secondary antibodies were used at 1:500 dilution in TBP. Secondary antibody staining was carried out for ~24 h at 4°C. Coverslips were rinsed twice in PBS with 50 mM glycine and once in deionized water, then air-dried and mounted on glass slides using fluorescent mounting medium. Confocal images of neuronal somata were acquired on a Leica Stellaris 5 microscope with a 63x oil-immersion objective. Single section, 92.65 x 92.65 μm^2^ areas were acquired using 2x digital zoom (1024 x 1024 pixel^2^, pixel size of 90.2 x 90.2 nm^2^). Imaging and laser settings were identical for all conditions within a given biological repeat. For analyses of somatic HA signals, the NeuN signal was used to mark the neuron somata, and EGFP-Cre or EGFP-ΔCre was used to define nuclei. Somatic ROIs were drawn as donut shapes by using the outer edge of the NeuN profile along the main somatic compartment not including neurites, and by excluding the EGFP-labeled nucleus. The average pixel intensity within the somatic ROI was then calculated for the HA channel and plotted for each cell. Representative images in figures were cropped and adjusted for brightness and contrast to facilitate inspection. Brightness and contrast adjustments were made for display in figures and were done identically for images within an experiment, but image quantification was performed on raw images without these adjustments. The experimenter was blind to the condition/genotype for image acquisition and analyses.

#### Electrophysiological recordings from neurons

Electrophysiological recordings in cultured hippocampal neurons were performed as established before^20,41,95^ at DIV16 to 19. Glass pipettes were pulled at 2 to 5 MΩ and filled with intracellular solution containing (in mM) for mEPSCs and EPSCs: 120 Cs-methane-sulfonate, 2 MgCl_2_, 10 EGTA, 4 Na_2_-ATP, 1 Na-GTP, 4 QX314-Cl, 10 HEPES-CsOH (pH 7.4, ~300 mOsm); and for mIPSCs and IPSCs: 40 CsCl, 90 K-gluconate, 1.8 NaCl, 1.7 MgCl_2_, 3.5 KCl, 0.05 EGTA, 2 Mg-ATP, 0.4 Na_2_-GTP, 10 phosphocreatine, 4 QX314-Cl, 10 HEPES-CsOH (pH 7.2, ~300 mOsm). The extracellular solution contained (in mM): 140 NaCl, 5 KCl, 2 MgCl_2_, 1.5 CaCl_2_, 10 glucose, 10 HEPES-NaOH (pH 7.4, −300 mOsm). Cells were held at +40 mV for NMDAR-EPSCs and at −70 mV for IPSCs, mIPSCs, and mEPSCs, and recordings were performed at room temperature (20°C–24°C). For recording of evoked synaptic currents, access resistance was compensated to 2–3 MΩ, and cells were discarded if the uncompensated access resistance exceeded 15 MΩ. For recordings of miniature synaptic currents, cells were discarded if the uncompensated access resistance exceeded 20 MΩ. The following drugs were added to the extracellular solution: for NMDAR-EPSCs, picrotoxin (PTX, 50 μM) and 6-Cyano-7-nitroquinoxaline-2,3-dione (CNQX, 20 μM); for IPSCs, D-2-amino-5-phosphonopentanoic acid (D-AP5, 50 μM) and CNQX (20 μM); for mEPSCs, TTX (1 μM), PTX (50 μM), and D-AP5 (50 μM); and for mIPSCs, TTX (1 μM), D-AP5 (50 μM), and CNQX (20 μM). Action potentials were elicited with a bipolar focal stimulation electrode fabricated from nichrome wire. To evaluate the Ca_V_ blocker sensitivity of synaptic transmission, ω-agatoxin IVA (to block Ca_V_2.1) or isradipine (to block Ca_V_1s) were used. Blockers were pipetted into the recording chamber as stocks diluted in extracellular solution for a final working concentration of 200 nM ω-agatoxin IVA and 20 μM isradipine. For wash-in, cells were incubated after blocker addition for 5 min. IPSCs were recorded first in the absence of Ca_V_ blockers. Then, IPSCs were measured after wash-in of 200 nM ω-agatoxin IVA and again after wash-in of 200 nM ω-agatoxin IVA and 20 μM isradipine (Figures 4F-4I), or after wash-in of 20 μM isradipine (Figures S4H-S4J). Data were acquired at 10 kHz and lowpass filtered at 2 kHz with an Axon 700B Multiclamp amplifier and digitized with a Digidata 1440A digitizer. Data acquisition and analyses were done using pClamp10. Spontaneous mEPSCs and mIPSCs were identified with a template search and individually cross-checked. Frequencies were obtained for each cell by dividing the event count by the recording duration, and amplitudes were quantified per cell by extracting the peak amplitude of each event followed by averaging. For electrophysiological experiments, the experimenter was blind to the genotype throughout data acquisition and analyses.

#### Western blotting

Lysates from transfected HEK293T cells were used for Western blotting. Ca_V_1 and Ca_V_2 constructs were co-transfected with Ca_V_β1b (p754; pMT2 Ca_V_β1b-GFP was a gift from A. Dolphin, Addgene plasmid # 89893; http://n2t.net/addgene:89893; RRID: Addgene_89893)^36^ and Ca_V_α2δ1 (p752; Ca_V_α2δ1 was a gift from D. Lipscombe, Addgene plasmid # 26575; http://n2t.net/ addgene:26575; RRID: Addgene_26575).^84^ Plasmids were transfected with the Ca^2+^ phosphate method at a 1:1:1 molar ratio with a total of 6.7 μg of DNA. Around 48 h after transfection, HEK293T cells were harvested in 1 mL of standard 1x SDS buffer per flask. Homogenates were centrifuged at 16,200 x g for 10 min at room temperature, run on 6% (for Ca_V_s) or 12% (for β-actin) polyacrylamide gels, and transferred onto nitrocellulose membranes for 6.5 h at 4°C in transfer buffer (containing per L, 200 mL methanol, 14 g glycine, 3 g Tris). Membranes were blocked in filtered 10% nonfat milk/5% goat serum in TBST (Tris-buffered saline with 0.1% Tween) for 1 h at room temperature and incubated with primary antibodies in 5% nonfat milk/2.5% goat serum in TBST overnight at 4°C. The primary antibodies used were mouse IgG1 anti-HA (1:1000; RRID: AB_2565006, A12) and mouse IgG1 anti-β-actin (1:2000; RRID: AB_476692, A127). Membranes were washed five times for 3 min each at room temperature in TBST and then incubated with secondary antibodies in 5% nonfat milk/2.5% goat serum in TBST for 1 h at room temperature. The secondary antibodies used were peroxidase-conjugated goat anti-mouse IgG (1:10,000, RRID: AB_2334540, S52) and peroxidase-conjugated goat anti-rabbit IgG (1:10,000, RRID: AB_2334589, S53). Membranes were again washed five times for 3 min each at room temperature in TBST, then incubated in a chemiluminescent reagent for 30 s. Finally, the membranes were exposed to films, and films were developed and scanned. Corresponding western blots of Ca_V_s and β-actin were run simultaneously, on the same day, and on separate gels using the same samples. For illustration in figures, blots were rotated with bilinear interpolation and cropped for display.

#### Electrophysiological recordings in HEK293T cells

Electrophysiological recordings in HEK293T cells were performed as established before.^20^ HEK293T cells plated on 12 mm diameter coverslips were transfected by the Ca^2+^ phosphate method with Ca_V_β1 (p754), Ca_V_α2Δ1 (p752), and a Ca_V_α1 subunit. Constructs were transfected at a 1:1:1 molar ratio with a total of 1 mg of DNA per coverslip. Current recordings were carried out 48 h after transfection at room temperature (20°C–24°C). The extracellular solution contained (in mM): 10 BaCl_2_, 140 TEA-Cl, 10 HEPES, and 10 glucose (7.4 pH with TEA-OH). Glass pipettes were pulled at 2 to 5 MU and filled with intracellular solution containing (in mM): 135 Cs-methanesulfonate, 5 CsCl, 0.5 EGTA, 5 MgCl_2_, 4 Na_2_-ATP, and 10 HEPES (pH7.2 with CsOH). Whole cell recordings were performed on HEK293T cells with robust GFP expression, and cells were discarded if the access resistance exceeded 15 MΩ. To characterize the current-voltage relationship, cells were held at −80 mV, and inward currents were evoked by 50 ms depolarizations from −70 mV to +50 mV in increments of 10 mV. The maximal current during each depolarization step was normalized to the overall peak inward current to generate a normalized I-V curve. Only cells that had a peak inward current greater than 300 pA at any voltage step were included in the analysis. Data were acquired with an Axon 700B Multiclamp amplifier and digitized with a Digidata 1440A digitizer. Pulse-noise leak subtraction was used to isolate active currents, and no resistance compensation was applied. The experimenter was blind to the genotype throughout data acquisition and analyses.

### QUANTIFICATION AND STATISTICAL ANALYSES

Data are displayed as mean ± SEM. Individual data points are displayed as open circles for datasets with sample sizes below 100. Violin plots were used for datasets with sample sizes above 100. Statistics were performed in GraphPad Prism 9, and significance is presented as *p < 0.05, **p < 0.01, and ***p < 0.001. Sample sizes and statistical tests for each experiment are included in each figure legend. For electrophysiological experiments, the sample size used for statistical analyses was the number of recorded cells. For STED microscopic data, the sample size used for statistical analyses was the number of synapses. For confocal microscopic data, the sample size used for statistical analyses was the number of images for analyses of synapsin ROIs, or the number of neurons for analyses of somata. Non-parametric tests that do not make assumptions of data normality and homoscedasticity were used whenever possible to compare genotypes or experimental conditions. Single factor, multiple group comparisons were conducted using Kruskal-Wallis tests followed by Dunn’s multiple comparisons post-hoc tests for proteins of interest (HA or Ca_V_2.1), for current amplitudes (EPSCs, IPSCs), and for event frequencies (mEPSCs, mIPSCs). To compare the efficacy of blockade of synaptic transmission by different pharmacological agents in Figure 4H, Friedman tests and Dunn’s multiple comparisons post-hoc tests were used. To compare the effects of Ca_V_ blockers on synaptic transmission across genotypes in Figures 4I, a two-way, repeated-measures ANOVA and Dunnett’s multiple comparisons post-hoc tests was used. To compare the current-voltage relationship for multiple Ca_V_ constructs in Figure S4C, a two-way, repeated-measures ANOVA was used. To assess blocker sensitivity in Figure S4J, the Wilcoxon matched-pairs signed rank test was used.

## Supplementary Material

1

## Figures and Tables

**Table:** KEY RESOURCES TABLE

REAGENT of RESOURCE	SOURCE	IDENTIFIER
Antibodies		
rabbit anti-Ca_V_2.1 (lab antibody code (LAC): A46)	Synaptic Systems	Cat#: 152203 RRID: AB_2619841
mouse IgG1 anti-HA (LAC: A12)	Biolegend	Cat#: 901501 RRID: AB_2565006
guinea pig anti-PSD-95 (LAC: A5)	Synaptic Systems	Cat#: 124014 RRID: AB_2619800
rabbit anti-synapsin (LAC: A30)	Abcam	Cat#: ab8 RRID: AB_2200097
mouse IgG1 anti-synapsin (LAC: A57)	Synaptic Systems	Cat#: 106001 RRID: AB_2617071
mouse IgG2b anti-NeuN (LAC: A254)	Abcam	Cat#: ab104224 RRID: AB_10711040
mouse IgG1 anti-β-actin (LAC: A127)	Sigma	Cat#: A1978 RRID: AB_476692
goat anti-mouse IgG1 Alexa Fluor 488 (LAC: S7)	ThermoFisher	Cat#: A-21121 RRID: AB_2535764
goat anti-guinea pig Alexa Fluor 555 (LAC: S23)	ThermoFisher	Cat#: A-21435 RRID: AB_2535856
goat anti-rabbit Alexa Fluor 633 (LAC: S33)	ThermoFisher	Cat#: A-21070 RRID: AB_2535731
goat anti-rabbit Alexa Fluor 488 (LAC: S5)	ThermoFisher	Cat#: A-11034 RRID: AB_2576217
goat anti-mouse IgG1 Alexa Fluor 633 (LAC: S29)	ThermoFisher	Cat#: A-21126 RRID: AB_2535768
goat anti-mouse IgG1 Alexa Fluor 555 (LAC: S19)	ThermoFisher	Cat#: A-21127 RRID: AB_2535769
goat anti-mouse IgG2b Alexa Fluor 633 (LAC: S31)	ThermoFisher	Cat#: A-21146 RRID: AB_1500899
goat anti-mouse peroxidase-conjugated (LAC: S52)	MP Biomedicals	Cat#: 0855550 RRID: AB_2334540
goat anti-rabbit peroxidase-conjugated (LAC: S53)	MP Biomedicals	Cat#: 0855676 RRID: AB_2334589
Chemicals, Peptides and Recombinant Proteins		
Picrotoxin (PTX)	Tocris	Cat#: 1128
D-2-amino-5-phosphonopentanoic acid (D-AP5)	Tocris	Cat#: 0106
6-Cyano-7-Nitroquinoxaline-2,3-dione (CNQX)	Tocris	Cat#: 0190
QX314-Cl	Tocris	Cat#: 2313
ω-Agatoxin IVA	Alomone	Cat#: STA-500
Isradipine	Tocris	Cat#: 2004
Tetrodotoxin (TTX)	Tocris	Cat#: 1078
Experimental Models: Cell Lines		
HEK293T	ATCC	Cat#: CRL-3216 RRID:CVCL_0063
Experimental Models: Organisms/Strains		
Ca_V_2.1 (*Cacna1a*) conditional knockout	Todorov et al.^87^	N/A
Ca_V_2.2 (*Cacna1b*) conditional knockout	Held et al.^20^	Cat# KOMP: CSD34514 RRID: MMRRC_046864-UCD
Ca_V_2.3 (*Cacna1e*) conditional knockout	Pereverzev et al.^88^	N/A
Recombinant DNA		
pFSW EGFP-ΔCre (lab plasmid code (LPC): p010)	Liu et al.^89^	N/A
pFSW EGFP-Cre (LPC: p009)	Liu et al.^89^	N/A
pREV (LPC: 023)	Liu et al.^89^	N/A
pRRE (LPC: 024)	Liu et al.^89^	N/A
pVSVG (LPC: 025)	Liu et al.^89^	N/A
pFSW (LPC: 008)	Liu et al.^89^	N/A
pCMV HA-Ca_V_2.1 (LPC: p771)	Held et al.^20^	N/A
pCMV HA-Ca_V_2.1^ΔEF1^ (LPC: p939)	this study	N/A
pCMV HA-Ca_V_1.3 (LPC: p1073)	this study	N/A
pCMV HA-Ca_V_1.3^2.1Ct^ (LPC: p1074)	this study	N/A
pCMV HA-Ca_V_2.1^1.3Ct^ (LPC: p1075)	this study	N/A
pCMV HA-Ca_V_1.3^ΔCt^ (LPC: p1076)	this study	N/A
pCMV HA-Ca_V_1.3^2.1ProxCt^ (LPC: p1081)	this study	N/A
pCMV HA-Ca_V_1.3^2.1DistCt^ (LPC: p1082)	this study	N/A
pMT2 Ca_V_β1b-GFP (LPC: p754)	Page et al.^36^	Cat#: 89893 RRID: Addgene_89893
pCNDA3.1 Ca_V_α2δ-1 (LPC: p752)	Lin et al.^84^	Cat#: 26575 RRID: Addgene_26575
pFSW HA-Ca_v_2.1 (LPC: p789)	Held et al.^20^	N/A
pFSW HA-Ca_V_2.1^ΔEF1^ (LPC: p947)	this study	N/A
pFSW HA-Ca_V_1.3 (LPC: p1077)	this study	N/A
pFSW HA-Ca_V_1.3^2.1Ct^ (LPC: p1078)	this study	N/A
pFSW HA-Ca_V_2.1^1.3Ct^ (LPC: p1079)	this study	N/A
pFSW HA-Ca_V_1.3^ΔCt^ (LPC: p1080)	this study	N/A
pFSW HA-Ca_V_1.3^2.1ProxCt^ (LPC: p1083)	this study	N/A
pFSW HA-Ca_V_1.3^2.1DistCt^ (LPC: p1084)	this study	N/A
Oligonucleotides		
*Cacna1a* forward primer: ACCTACAGTCTGCCAGGAG	Held et al.^20^	N/A
*Cacna1a* reverse primer: TGAAGCCCAGACATCCTTGG	Held et al.^20^	N/A
*Cacna1b* forward primer: TGGTTGGTGTCCTGTTCTCC	Held et al.^20^	N/A
*Cacna1b* reverse primer: TAAGGAGCAGGGAATCCTGG	Held et al.^20^	N/A
*Cacna1e* forward primer: GACAAGACCCCAATGTCTCG	Held et al.^20^	N/A
*Cacna1e* reverse primer: TCCATGTTCCTTCTCACTCC	Held et al.^20^	N/A
Software and Algorithms		
Fiji	Schindelin et al.^90^	RRID: SCR_00228 https://imagej.net/Fiji/Downloads
Prism9	GraphPad	RRID: SCR_002798 https://www.graphpad.com/scientific-software/prism
pClamp	Molecular Devices	RRID: SCR_011323 https://www.moleculardevices.com/products/software/pclamp.html
MATLAB	MathWorks	RRID: SCR_001622 https://www.mathworks.com/products/matlab.html
MATLAB code for automatic object detection	Tan et al.^95^	https://doi.org/10.5281/zenodo.6388196

## Highlights

- Ca_V_2.1 s, but not Ca_V_1.3s, are at the active zone and trigger neurotransmitter release
- Swapping the Ca_V_2.1 C-terminus onto Ca_V_1.3 confers active zone targeting to Ca_V_1.3
- Ca_V_1.3 with the Ca_V_2.1 C-terminus mediates release that is L-type blocker sensitive
- The first EF hand in the C-terminus is required for Ca_V_2.1 active zone targeting
