# Supplementary material for: The intracellular C-terminus confers compartment-specific targeting of voltage-gated calcium channels
Source: Cell Rep. Author manuscript; Available in PMC 2024 Sep 30. (PMC11441329; doi:10.1016/j.celrep.2024.114428)
Supplement: 1 [file NIHMS2011880-supplement-1.pdf]

Cell Reports, Volume *43*

Supplemental information

The intracellular C-terminus confers compartment-specific targeting of voltage-gated calcium channels

Morven Chin and Pascal S. Kaeser

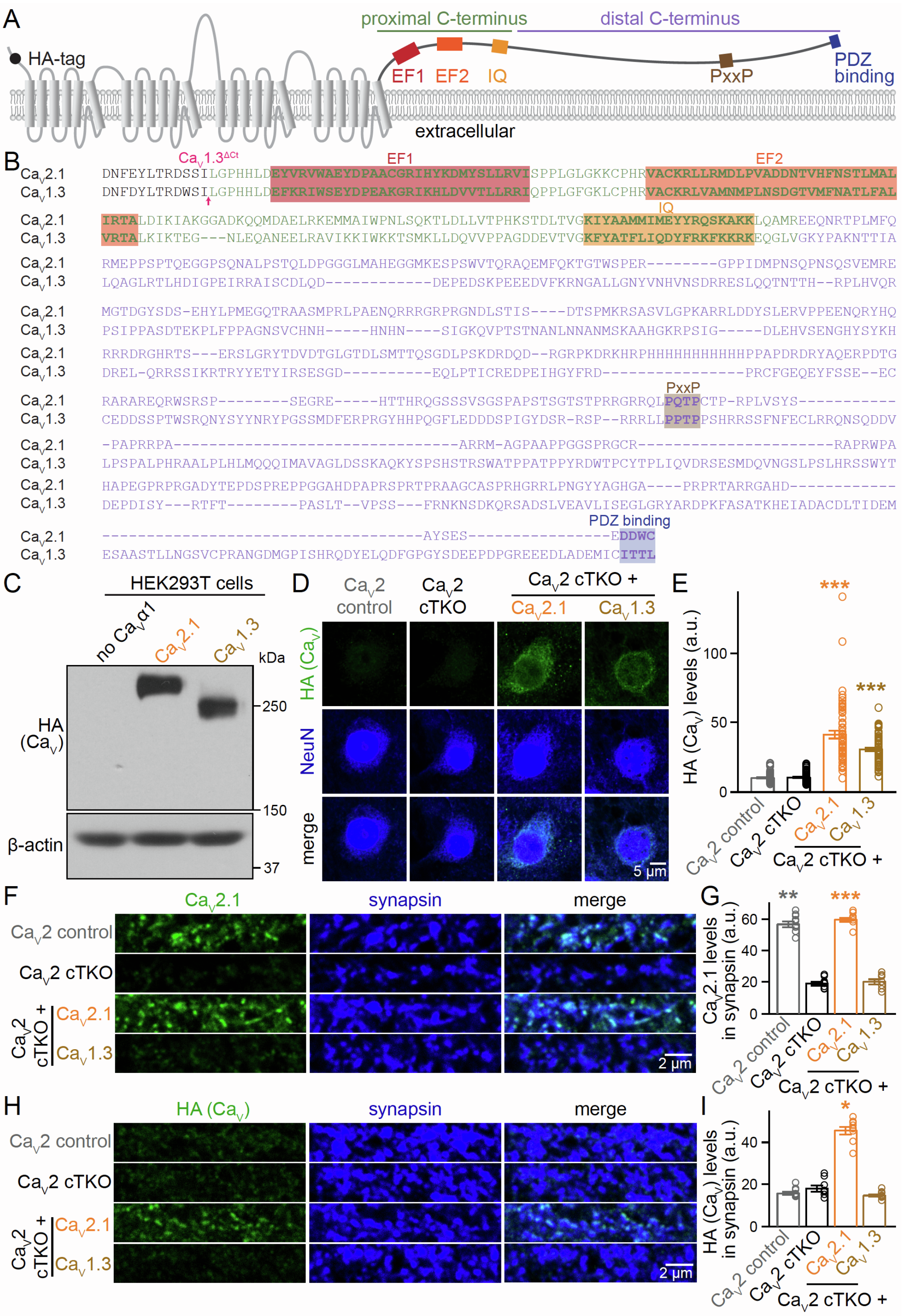

Figure S1. Additional assessment of Ca$_V$2.1 and Ca$_V$1.3 expression and localization, related to Figure 1.

(A) Schematic of $Ca_V2.1$ with conserved sequence motifs highlighted, adapted from [20]; EF1 and EF2: EF hands; IQ: IQ motif; PxxP: proline rich motif.

(B) Alignment of the C-terminal sequences starting immediately after the last transmembrane segment (for $Ca_V2.1$, residues DNFE…DDWC are matching with GenBank Entry AY714490.1; for $Ca_V1.3$, residues DNFD…ITTL are matching with GenBank Entry AF370010.1). Sequence motifs that are conserved between Ca_V2s and/or Ca_V1s are highlighted, and the Ca_V proximal and distal C-terminal segments are labeled in green and purple, respectively.

(C) Western blot of HEK293T cell homogenates after transfection with $Ca_V\beta1$, $Ca_V\alpha2\delta1$, and without (no $Ca_V\alpha1$) or with a $Ca_V\alpha1$ subunit to assess $Ca_V\alpha1$ expression; $Ca_V2.1$ and $Ca_V1.3$ were transfected and analyzed multiple times, but only once in this order.

(D+E) Representative confocal images (D) and quantification (E) of HA levels in cell bodies of neurons stained with antibodies against HA and NeuN. Cell bodies were defined as donut shaped ROIs using the outer edge of the NeuN profile along the main somatic compartment not including the neurites, and by excluding the EGFP-labeled nucleus; 60 somata/3 cultures each.

(F+G) Representative areas of confocal images (F) and quantification (G) of $Ca_V2.1$ levels in synapsin ROIs (the imaged areas are identical to the STED scans in Fig. 1C-E); Ca_V2 control, 9 images/3 cultures; Ca_V2 cTKO, 8/3; Ca_V2 cTKO + $Ca_V2.1$, 9/3; Ca_V2 cTKO + $Ca_V1.3$, 8/3.

(H+I) As in F and G, but for neurons stained with antibodies against HA, PSD-95 and synapsin (the imaged areas are identical to the STED scans in Fig. 1F-H); Ca_V2 control, 9/3; Ca_V2 cTKO, 8/3; Ca_V2 cTKO + $Ca_V2.1$, 9/3; Ca_V2 cTKO + $Ca_V1.3$, 9/3.

Data are mean ± SEM; *p < 0.05, **p < 0.01, and ***p < 0.001. Statistical significance compared to Ca_V2 cTKO was determined with Kruskal-Wallis tests followed by Dunn's multiple comparisons post-hoc tests for the proteins of interest in E, G, and I.

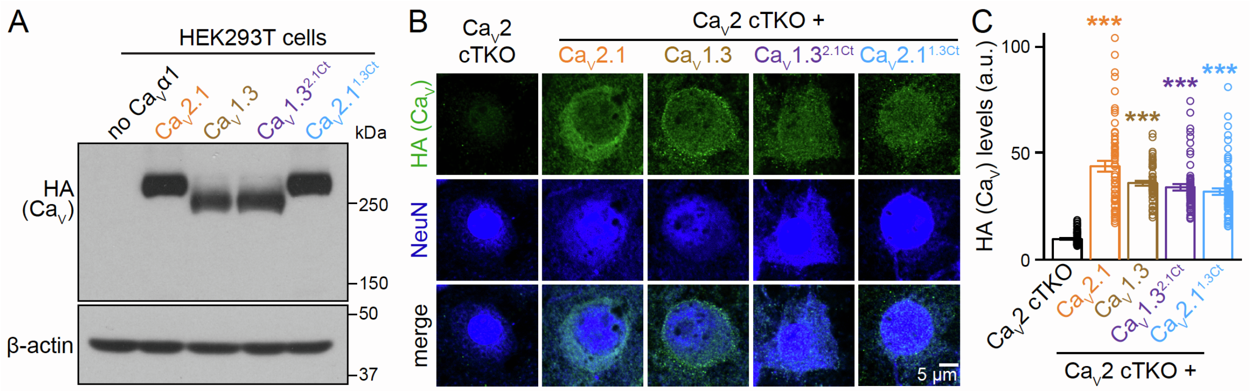

Figure S2. Additional assessment of $Ca_V1.3^{2.1Ct}$ and $Ca_V2.1^{1.3Ct}$ expression, related to Figure 2.

(A) Western blot of HEK293T cell homogenates after transfection with $Ca_V\beta1$, $Ca_V\alpha2\delta1$, and without (no $Ca_V\alpha1$) or with a $Ca_V\alpha1$ subunit to assess $Ca_V\alpha1$ expression, a representative blot from three independent repeats is shown.

(B+C) Representative confocal images (B) and quantification (C) of HA levels in cell bodies of neurons stained with antibodies against HA and NeuN; 60 somata/3 cultures each.

Data are mean ± SEM; ***$p < 0.001$. Statistical significance compared to Ca_V2 cTKO was determined with Kruskal-Wallis tests followed by Dunn's multiple comparisons post-hoc tests for the protein of interest in C.

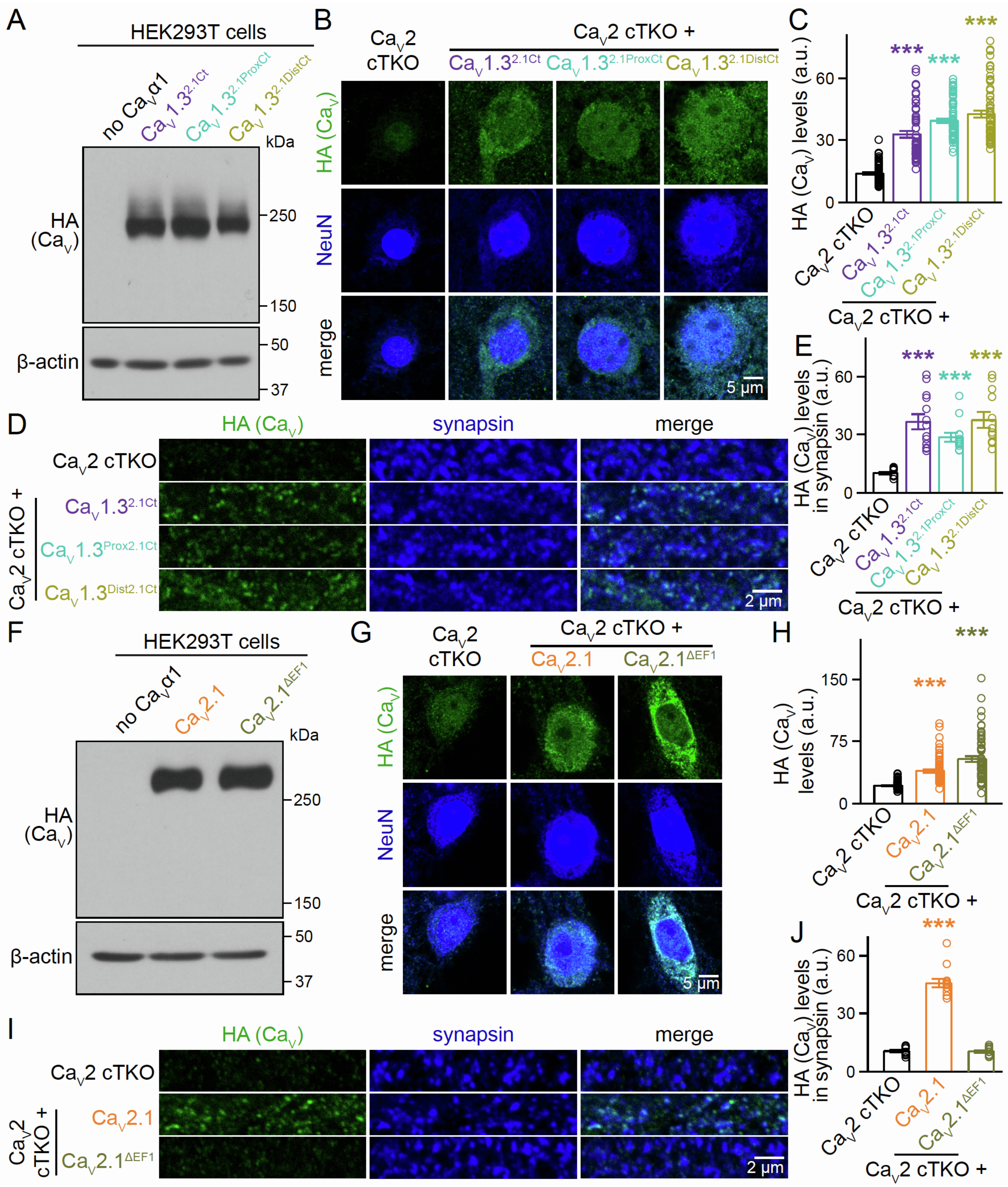

Figure S3. Additional assessment of Ca$_V$1.3$^{2.1ProxCt}$, Ca$_V$1.3$^{2.1DistCt}$ and Ca$_V$2.1^{ΔEF1} expression and localization, related to Figure 3.

(A) Western blot of HEK293T cell homogenates after transfection with Ca$_V$β1, Ca$_V$α2δ1, and

without (no $Ca_V\alpha1$) or with a $Ca_V\alpha1$ subunit to assess $Ca_V\alpha1$ expression, a representative blot from three independent repeats is shown.

(B+C) Representative confocal images (B) and quantification (C) of HA levels in cell bodies of neurons stained with antibodies against HA and NeuN; 60 somata/3 cultures each.

(D and E) Representative areas of confocal images (D) and quantification (E) of HA levels in synapsin ROIs (the imaged areas are identical to the STED scans in Fig. 3B-D); Ca_V2 cTKO, 12 images/3 cultures; Ca_V2 cTKO + $Ca_V1.3^{2.1Ct}$, 13/3; Ca_V2 cTKO + $Ca_V1.3^{2.1ProxCt}$, 12/3; Ca_V2 cTKO + $Ca_V1.3^{2.1DistCt}$, 12/3.

(F) Western blot of HEK293T cell homogenates after transfection with $Ca_V\beta1$, $Ca_V\alpha2\delta1$, and without (no $Ca_V\alpha1$) or with a $Ca_V\alpha1$ subunit to assess expression, a representative blot from two independent repeats is shown.

(G+H) Representative confocal images (G) and quantification (H) of HA levels in cell bodies of neurons stained with antibodies against HA and NeuN; 60/3 each.

(I and J) Representative areas of confocal images (I) and quantification (J) of HA levels in synapsin ROIs (the imaged areas are identical to the STED scans in Fig. 3F-H); 12/3 each.

Data are mean ± SEM; $^{***}p < 0.001$. Statistical significance compared to Ca_V2 cTKO was determined with Kruskal-Wallis tests followed by Dunn's multiple comparisons post-hoc tests for the protein of interest in C, E, H, and J.

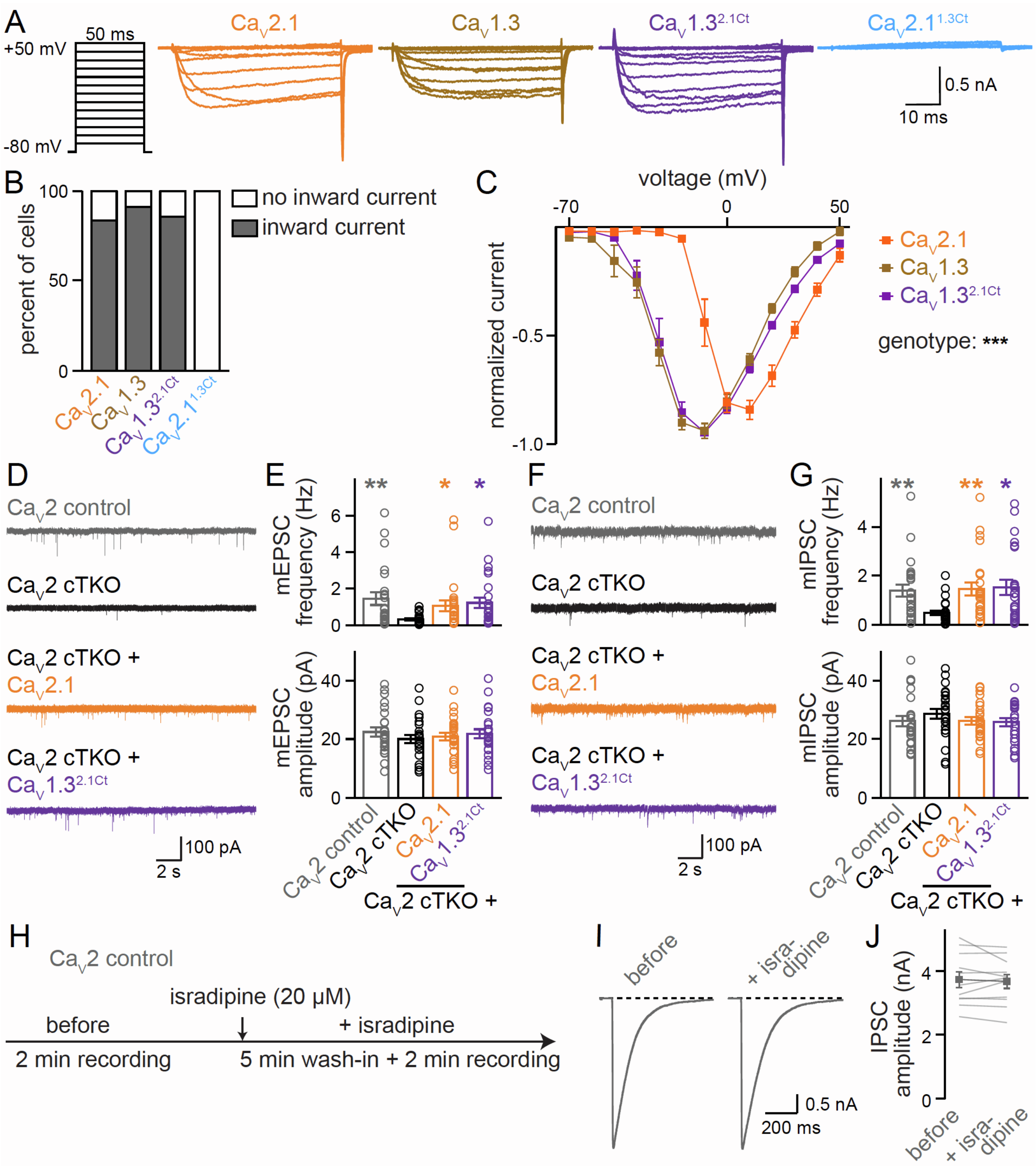

Figure S4. Additional electrophysiological experiments, related to Figure 4.

(A-C) Voltage-step protocol and representative currents (A), percentage of recorded HEK293T cells with inward currents (B), and summary plot (C) of the normalized I-V relationship for HEK293T cells transfected with $Ca_V\beta1$, $Ca_V\alpha2\delta1$, and a $Ca_V\alpha1$ construct, B: $Ca_V2.1$, 26 cells/3

transfections; $Ca_V1.3$, 25/3; $Ca_V1.3^{2.1Ct}$, 30/3; $Ca_V2.1^{1.3Ct}$, 23/3; C: $Ca_V2.1$, 13/3; $Ca_V1.3$, 13/3; $Ca_V1.3^{2.1Ct}$, 11/3; in C, cells from B with a peak inward current smaller than 300 pA were not included.

(D+E) Representative traces (D) and quantifications (E) of miniature EPSC frequency and amplitude; 24 cells/3 cultures each.

(F+G) As in D and E, but for miniature IPSCs, 24/3 each.

(H-J) Experimental strategy (H), representative traces (I) and quantification (J) of IPSCs recorded in CaV2 control neurons before and after the addition of 20 μM isradipine; 11 cells/3 cultures.

Data are mean ± SEM; and *p < 0.05, **p < 0.01, ***p < 0.001. Statistical significance compared to $Ca_V1.3$ was determined with two-way ANOVA in C (p < 0.001 for genotype, < 0.001 for voltage, < 0.001 for interaction). Statistical significance compared to Ca_V2 cTKO was determined with Kruskal-Wallis tests followed by Dunn's multiple comparisons post-hoc tests for the frequency of spontaneous miniature excitatory and inhibitory events in E and G.

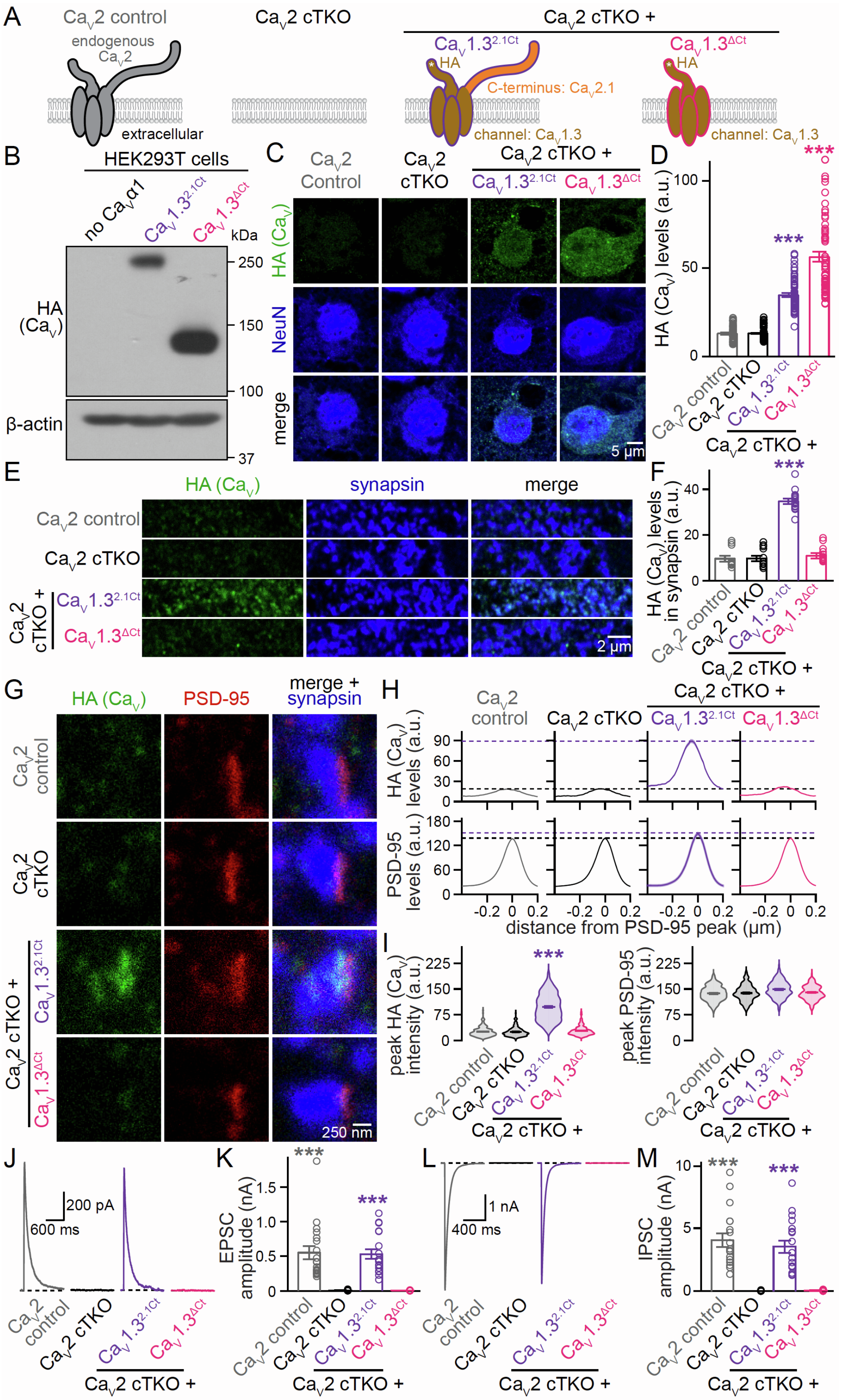

Figure S5. Assessment of $Ca_V1.3^{\Delta Ct}$, related to Figure 4.

(A) Schematic of the conditions for comparison.

(B) Western blot of HEK293T cell homogenates after transfection with $Ca_V\beta1$, $Ca_V\alpha2\delta1$, and without (no $Ca_V\alpha1$) or with a $Ca_V\alpha1$ subunit to assess $Ca_V\alpha1$ expression, a representative blot from two independent repeats is shown.

(C+D) Representative confocal images (C) and quantification (D) of HA levels in cell bodies of neurons stained with antibodies against HA and NeuN; 60 somata/3 cultures each.

(E+F) Representative areas of confocal images (E) and quantification (F) of HA levels in synapsin ROIs; Ca_V2 control, 11 images/3 cultures; Ca_V2 cTKO, 12/3; Ca_V2 cTKO + $Ca_V1.3^{2.1Ct}$, 14/3; Ca_V2 cTKO + $Ca_V1.3^{\Delta Ct}$, 14/3.

(G-I) Representative images (G) and summary plots of intensity profiles (H) and peak levels (I) of HA and PSD-95 at side-view synapses stained for HA (STED), PSD-95 (STED), and synapsin (confocal). The imaged areas are identical to the ones used for confocal analyses in E+F. Dashed lines in H denote levels in Ca_V2 cTKO (black) and Ca_V2 cTKO + $Ca_V1.3^{2.1Ct}$ (purple); Ca_V2 control, 198 synapses/3 cultures; Ca_V2 cTKO, 190/3; Ca_V2 cTKO + $Ca_V1.3^{2.1Ct}$, 207/3; Ca_V2 cTKO + $Ca_V1.3^{\Delta Ct}$, 195/3.

(J+K) Representative traces (J) and quantification (K) of NMDAR-mediated EPSCs; 18 cells/3 cultures each.

(L+M) As in J and K, but for IPSCs; 18/3 each.

Data are mean ± SEM; and ***$p < 0.001$. Statistical significance compared to Ca_V2 cTKO was determined with Kruskal-Wallis tests followed by Dunn's multiple comparisons post-hoc tests for the protein of interest or amplitudes in D, F, I, K, and M.
